# Association of Increased Prostate-Specific Antigen Levels After Treatment and Mortality in Men With Locally Advanced vs Localized Prostate Cancer: A Secondary Analysis of 2 Randomized Clinical Trials

**DOI:** 10.1001/jamanetworkopen.2021.11092

**Published:** 2021-05-17

**Authors:** Martin T. King, Ming-Hui Chen, Laurence Collette, Anouk Neven, Michel Bolla, Anthony V. D’Amico

**Affiliations:** 1 Department of Radiation Oncology, Dana-Farber Cancer Institute, Brigham and Women’s Hospital, Boston, Massachusetts; 2 Department of Statistics, University of Connecticut, Storrs; 3 European Organisation for Research and Treatment of Cancer Headquarters, Brussels, Belgium; 4 Department of Radiation Oncology, Grenoble University Hospital, Grenoble, France

## Abstract

**Importance:**

Increased prostate-specific antigen (PSA) levels after treatment (PSA failure) may have different associations with outcomes for men with locally advanced vs localized prostate cancer.

**Objective:**

To evaluate whether the association between PSA failure and death may be different in locally advanced vs localized prostate cancer.

**Design, Setting, and Participants:**

This multicenter cohort study included patients from 2 randomized clinical trials. The Dana-Farber Cancer Institute (DFCI) 95-096 trial randomized 206 men with localized prostate cancer from December 1, 1995, to April 15, 2001, whereas the European Organisation for Research and Treatment of Cancer (EORTC) 22961 trial randomized 970 men with locally advanced prostate cancer from October 30, 1997, to May 1, 2002. Data were analyzed from January 1, 2020, to October 31, 2020.

**Interventions:**

The DFCI 95-096 trial randomized men to 0 vs 6 months of androgen deprivation therapy (ADT) with external beam radiotherapy; the EORTC 22961 trial randomized men to 6 vs 36 months of ADT with external beam radiotherapy.

**Main Outcomes and Measures:**

For each trial, the PSA doubling time (time to doubling of PSA levels) associated with PSA failure was evaluated. The risk of all-cause mortality associated with PSA failure (nadir plus 2 definition) was evaluated after adjustment of baseline covariates and treatment.

**Results:**

This analysis included a total of 1173 men (206 from DFCI 95-096 and 967 with available tumor stage from EORTC 22961; median age, 70.0 [interquartile range (IQR), 65.0-74.0 years). For DFCI 95-096, 161 men died (30 [18.6%] due to prostate cancer) at a median follow-up of 18.2 (IQR, 17.3-18.8) years. Among the 108 men with PSA failure, the median PSA doubling time was 13.0 (IQR, 7.4-31.1) months. For EORTC 22961, 230 men died (75 [32.6%] due to prostate cancer) at a median follow-up of 6.4 (IQR, 6.3-6.6) years. Among 290 men who experienced PSA failure, the median PSA doubling time was 5.0 (IQR, 2.9-8.9) months. Compared with DFCI 95-096, PSA failure was associated with a higher risk of all-cause mortality in EORTC 22961 (adjusted hazard ratios, 3.98 [95% CI, 2.92-5.44]; *P* < .001 vs 1.51 [95% CI, 1.03-2.23]; *P* = .04).

**Conclusions and Relevance:**

The association of PSA failure with outcomes may differ between locally advanced and localized prostate cancer. This finding supports the study of treatment intensification with the use of novel antiandrogen agents in addition to ADT at the time of PSA failure after treatment for locally advanced disease.

**Trial Registration:**

ClinicalTrials.gov Identifiers: NCT00116220 and NCT00003026

## Key Points

**Question:**

Is the association between increased prostate-specific antigen levels after treatment (PSA failure) and the risk of death different in men with locally advanced compared with localized prostate cancer?

**Findings:**

In this cohort study of men from 2 randomized clinical trials, PSA failure was associated with a higher risk of death in a trial of locally advanced cancer compared with a trial of localized prostate cancer.

**Meaning:**

These findings suggest that PSA failure may be associated with a worse outcome in men who have locally advanced rather than localized prostate cancer.

## Teaser

This cohort study evaluates whether the association between prostate-specific antigen (PSA) failure and death may be different in men with locally advanced vs localized prostate cancer enrolled in 2 randomized clinical trials.

## Introduction

The success of definitive external beam radiotherapy (EBRT) for prostate cancer is often measured via control of prostate-specific antigen (PSA) levels.^1,2^ This occurs largely because PSA tests are inexpensive and widely available and provide an early indicator of treatment response. In fact, increased PSA levels after treatment (PSA failure) have been incorporated as a primary end point in multiple phase 3 randomized clinical trials.^3,4,5^ Furthermore, current National Cancer Center Network guidelines recommend measurement of PSA levels as surveillance after definitive EBRT.^6^

However, most patients who experience PSA failure do not die of prostate cancer. In a recent analysis of the Dana-Farber Cancer Institute (DFCI) 95-096 trial, only 29 of 108 men (26.9%) who experienced PSA failure died of prostate cancer at a median follow-up of 16.6 (interquartile range [IQR], 15.4-17.7) years.^7^ Furthermore, a recent meta-analysis from the Intermediate Clinical Endpoints in Cancer of the Prostate (ICECaP) working group reported that PSA event–free survival exhibited a weak association with overall survival.^8^

Although PSA failure may not be a strong surrogate for survival, it may be associated with worse outcomes for certain patient populations. For example, in an analysis of the DFCI 95-096 trial, PSA failure was associated with increased all-cause mortality (ACM) in men with no or minimal comorbidity, but not moderate or severe comorbidity.^7^ These results suggest that men with significant competing risks who experience PSA failure may derive less of a survival benefit from salvage therapy compared with men with no or minimal comorbidities.

Likewise, men with locally advanced prostate cancer who experience PSA failure may be at greater risk of death compared with men with localized prostate cancer. The reason is that locally advanced disease may harbor more biologically aggressive cancer cells that may be more apt to metastasize after PSA failure. We herein evaluate whether the association between PSA failure and death may be different for men with localized vs locally advanced prostate cancer.

## Methods

We conducted an analysis of 2 mature randomized clinical trials in which the effects of treatment on survival have been analyzed: the DFCI 95-096 trial^9^ and the European Organisation for Research and Treatment of Cancer (EORTC) 22961 trial.^10^ CONSORT diagrams for both trials are shown in eFigures 1 and 2 in Supplement 1. All investigations were approved by the institutional review boards of the trial sites and followed the Strengthening the Reporting of Observational Studies in Epidemiology (STROBE) reporting guideline for cohort studies.^11^ Trial protocols are available in Supplement 2 (DFCI 95-096) and Supplement 3 (EORTC 22961). All patients provided written informed consent.

### DFCI 95-096

The DFCI 95-096 trial enrolled 206 men with clinically localized T1 to T2 disease who had a PSA level of 10 to 40 ng/mL (to convert to μg/L, multiply by 1.0), a Gleason score of at least 7, or endorectal magnetic resonance imaging evidence of extracapsular extension or seminal vesicle invasion at Harvard University–affiliated hospitals from December 1, 1995, to April 15, 2001.^9^ All men received EBRT, in which 70.2 Gy was delivered in 39 fractions. Men were randomized to 0 vs 6 months of androgen deprivation therapy (ADT) and followed up prospectively for PSA failure (nadir plus 2 definition)^2^ and death. The final cutoff date for data collection was October 9, 2016.^7^ All 206 men were included in this analysis.

### EORTC 22961

The EORTC 22961 trial enrolled men with cT1c to cT2b/pN1 to pN2 or cT2c to cT4/cN0 to cN2 disease at European centers from April 24, 1997, to September 28, 2001. Of the 1113 men who received 6 months of ADT along with EBRT (70 Gy delivered in 35 fractions), 970 without disease progression were subsequently randomized to 0 vs 30 months of adjuvant ADT (for respective totals of 6 vs 36 months of ADT) from October 30, 1997, to May 1, 2002. Men were followed up prospectively for PSA failure (PSA level ≥1.5 ng/mL and an increase in PSA level on 2 successive occasions at least 3 months apart), clinical progression, and death. The final cutoff date for data collection was September 4, 2007.^10^ For this analysis, 3 men were excluded owing to missing American Joint Committee on Cancer tumor stage. We retrospectively reclassified PSA failure using the nadir plus 2 definition, to maintain consistency with DFCI 95-096.^2^

### Statistical Analysis

#### Descriptive Statistics

For both trials, we categorized baseline covariates by the presence or absence of PSA failure. We used the Wilcoxon rank sum test for continuous covariates, the Fisher exact test for categorical covariates, and the log-rank test for follow-up time.

Prostate-specific antigen doubling time (time to doubling of PSA level) was calculated for all men with PSA failure. This metric used PSA values from the time of PSA level nadir to the time before salvage treatment and assumed first-order kinetics. If multiple PSA nadir values ≤0.1 ng/mL were observed in succession, the time of PSA nadir was set as the last date of a PSA value of 0.1 ng/mL or less.

#### Association of PSA Failure With Outcome

We evaluated the association of PSA failure with ACM. For each trial, we used univariable and multivariable Cox proportional hazards regression models. All variables evaluated in univariable models were included in multivariable models.

For DFCI 95-096, the variables included age, baseline PSA level (logarithm), clinical American Joint Committee on Cancer tumor stage (cT1 vs cT2), Gleason score (6-7 vs 8-10), Adult Comorbidity Evaluation 27 comorbidity score (none/minimal vs moderate/severe), treatment (EBRT alone vs EBRT plus 6 months of ADT), and PSA failure, coded as a time-dependent variable. We also included an interaction term between the comorbidity score and treatment, given its significance in prior studies.^9,12^

For EORTC 22961, the variables included age, baseline PSA level (logarithm), clinical tumor stage (cT1-cT2 vs cT3-cT4), clinical node stage (cN0 vs cN1), Gleason score (6-7 vs 8-10 vs unknown), World Health Organization performance status (0 vs 1-2), treatment (EBRT plus 6 months of ADT vs EBRT plus 36 months of ADT), and PSA failure (nadir plus 2), coded as a time-dependent variable. We included an interaction term between performance status and treatment to maintain consistency across trials. We also performed a sensitivity analysis in which we used the prospective PSA failure definition in univariable and multivariable Cox proportional hazards regression models for this trial.

#### Landmark Analysis

To evaluate whether earlier PSA failure may have a stronger association with ACM, we performed a landmark analysis with respect to time from randomization. For both trials, serial annual landmarks spanned years 1 to 6. At each landmark time, we used multivariable Cox proportional hazards regression analysis to evaluate the association of PSA failure with ACM after the landmark time. We defined PSA failure as a binary variable, with a value of 1 assigned for men who experienced PSA failure before the landmark time and 0 otherwise. Each regression model was adjusted by the same variables discussed above. Men who had died or were unavailable for follow-up before the landmark time were excluded. Adjusted hazard ratios (AHRs) were then plotted against landmark points for each trial.

All statistical analyses were conducted with R, version 3.6.2 (R Project for Statistical Computing) from January 1, 2020, to October 31, 2020. Two-sided *P* ≤ .05 was classified as statistically significant.

## Results

This analysis included a total of 1173 men (median age, 70.0 [IQR, 65.0-74.0] years): 206 from DFCI 95-096 and 967 with available tumor stage from EORTC 22961. Table 1 and Table 2 show descriptive statistics for DFCI 95-096 and EORTC 22961, respectively. For DFCI 95-096, 161 men died, including 30 (18.6%) due to prostate cancer, at a median follow-up of 18.2 (IQR, 17.3-18.8) years. One hundred eight men experienced PSA failure at a median time of 7.8 (IQR, 5.5-12.5) years. The group with PSA failure exhibited greater percentages of cT2 disease, Gleason scores of 8 to 10, and treatment with EBRT alone. The median PSA doubling time among men with PSA failure was 13.0 (IQR, 7.4-31.1) months. Similar percentages of men were alive in the groups with (23 [21.3%]) and without (22 [22.4%]) PSA failure at last follow-up.

**Table 1.** Clinical Features of the DFCI 95-096 Trial Population by the Absence or Presence of PSA Failure

Clinical factor	Study group (n = 206)^a^		*P* value^b^
	No PSA failure (n = 98)	PSA failure (n = 108)	
Age, median (IQR), y	73 (71-76)	72 (67-75)	.09
PSA level, median (IQR), ng/mL^c^	9.9 (6.2-13.2)	12.2 (8.1-18.1)	>.99
AJCC tumor category			.03
cT2	43 (43.9)	65 (60.2)	
cT1	55 (56.1)	43 (39.8)	
Gleason score			.03
8-10	10 (10.2)	23 (21.3)	
6-7	88 (89.8)	85 (78.7)	
Comorbidity			.47
Moderate/severe	26 (26.5)	23 (21.3)	
None/minimal	72 (73.5)	85 (78.7)	
Randomly assigned treatment arm			<.001
EBRT plus 6 mo ADT	69 (70.4)	33 (30.6)	
EBRT	29 (29.6)	75 (69.4)	
PSA doubling time before PSA failure, median (IQR), mo	NA	13.0 (7.4-31.1)	NA
Follow-up time, median (IQR), y	18.2 (17.2-19.5)	18.2 (17.3-18.8)	.40
Follow-up status			NA
Alive	22 (22.4)	23 (21.3)	
Cause of death			
Prostate cancer	0	30 (27.8)	
Cardiovascular disease	24 (24.5)	15 (13.9)	
Other	52 (53.1)	40 (37.0)	

Abbreviations: ADT, androgen deprivation therapy; AJCC, American Joint Committee on Cancer; DFCI, Dana-Farber Cancer Institute; EBRT, external beam radiotherapy; IQR, interquartile range; NA, not available; PSA, prostate-specific antigen.

^a^ Unless specified otherwise, data are expressed as No. (%) of patients.

^b^ The Wilcoxon rank sum test was used for age and PSA level; the Fisher exact test was used for T stage, Gleason score, comorbidity, and treatment arm; and the log-rank test was used for follow-up time.

^c^ SI conversion factor: To convert PSA to μg/L, multiply by 1.0.

**Table 2.** Clinical Features of the EORTC 22961 Trial Population by the Absence or Presence of PSA Failure (Nadir Plus 2)

Clinical factor	Study group (n = 967)^a^		*P* value^b^
	No PSA failure (n = 677)	PSA failure (n = 290)	
Age, median (IQR), y	70 (65-74)	68 (62-72)	<.001
PSA level, median (IQR), ng/mL^c^	16.6 (10.4-29.4)	22.2 (13.0-41.4)	<.001
AJCC tumor category			.98
cT3-T4	527 (77.8)	227 (78.3)	
cT2	145 (21.4)	61 (21.0)	
cT1	5 (0.7)	2 (0.7)	
AJCC nodal category			<.001
cN1	42 (6.2)	40 (13.8)	
cN0	635 (93.8)	250 (86.2)	
Gleason score			.007
8-10	112 (16.5)	73 (25.2)	
6-7	535 (79.0)	207 (71.4)	
Unknown	30 (4.4)	10 (3.4)	
Performance status			.71
1-2	106 (15.7)	42 (14.5)	
0	571 (84.3)	248 (85.5)	
Randomly assigned treatment arm			<.001
EBRT plus 36 mo ADT	399 (58.9)	86 (29.7)	
EBRT plus 6 mo ADT	278 (41.1)	204 (70.3)	
PSA doubling time before PSA failure, median (IQR), mo	NA	5.0 (2.9-8.9)	NA
Follow-up time, median (IQR), y	6.3 (6.2-6.5)	6.7 (6.5-7.2)	<.001
Follow-up status			NA
Alive	540 (79.8)	197 (67.9)	
Cause of death			
Prostate cancer	7 (1.0)	68 (23.4)	
Cardiovascular disease	46 (6.8)	10 (3.4)	
Other	84 (12.4)	15 (5.2)	

Abbreviations: ADT, androgen deprivation therapy; AJCC, American Joint Committee on Cancer; EBRT, external beam radiotherapy; EORTC, European Organisation for Research and Treatment of Cancer; IQR, interquartile range; NA, not available; PSA, prostate-specific antigen.

^a^ Unless specified otherwise, data are expressed as No. (%) of patients.

^b^ The Wilcoxon rank sum test was used for age and PSA level; the Fisher exact test was used for T stage, Gleason score, performance status, and treatment arm; and the log-rank test was used for follow-up time.

^c^ SI conversion factor: To convert PSA to μg/L, multiply by 1.0.

For EORTC 22961, 230 of 967 men died, including 75 (32.6%) due to prostate cancer, at a median follow-up of 6.4 (IQR, 6.3-6.6) years. A total of 290 men experienced PSA failure. The median time to PSA failure was not reached. The group with PSA failure was characterized by younger age (median, 68 [IQR, 62-72] vs 70 [IQR, 65-74] years) and higher baseline PSA levels (median, 22.2 [IQR, 13.0-41.4] vs 16.6 [IQR, 10.4-29.4] ng/mL). Greater percentages of men in the group with PSA failure vs those without PSA failure had positive nodes (40 of 290 [13.8%] vs 42 of 677 [6.2%]), Gleason score of 8 to 10 (73 of 290 [25.2%] vs 112 of 677 [16.5%]), and treatment with EBRT plus 6 months of ADT (204 of 290 [70.3%] vs 278 of 677 [41.1%]). The median PSA doubling time was 5.0 (IQR, 2.9-8.9) months. A lower percentage of men with PSA failure were alive (197 of 290 [67.9%] vs 540 of 677 [79.8%]) at last follow-up.

### Association of PSA Failure With Outcome

For DFCI 95-096 (Table 3), significant factors associated with ACM on univariable analysis included older age (HR, 1.07 [95% CI, 1.04-1.11] per year; *P* < .001), Gleason score of 8 to 10 (HR, 1.83 [95% CI, 1.22-2.74]; *P* = .003), EBRT plus 6 months of ADT for the subgroups with moderate/severe comorbidity (HR, 1.97 [95% CI, 1.10-3.54]; *P* = .02) and no/minimal comorbidity (HR, 0.68 [95% CI, 0.47-0.97]; *P* = .04), and PSA failure (HR, 1.50 [95% CI, 1.08-2.08]; *P* = .01). In the multivariable model, significant factors associated with ACM included age (AHR, 1.08 [95% CI, 1.04-1.11] per year; *P* < .001), EBRT plus 6 months of ADT for the subgroup with moderate/severe comorbidity (AHR, 2.83 [95% CI, 1.51-5.28]; *P* = .001), and PSA failure (AHR, 1.51 [95% CI, 1.03-2.23]; *P* = .04). Of note, there were significant interactions between comorbidity and treatment arm in both the univariable model (*P* = .002) and the multivariable model (*P* < .001).

**Table 3.** Univariable and Multivariable Cox Proportional Hazards Regression Models for All-Cause Mortality for the DFCI 95-096 Trial

Variable	No. of men	No. of deaths by cause				Univariable analysis		Multivariable analysis with PSA failure (nadir plus 2)	
		All	Prostate cancer	CVD	Other	HR (95% CI)	*P* value	AHR (95% CI)	*P* value
Age, y	206	161	30	39	92	1.07 (1.04-1.11)	<.001	1.08 (1.04-1.11)	<.001
Logarithm of PSA level, ng/mL^a^	206	161	30	39	92	1.10 (0.87-1.38)	.42	1.14 (0.90-1.44)	.28
AJCC clinical tumor category									
cT2	108	81	21	19	41	0.93 (0.68-1.27)	.65	0.82 (0.59-1.14)	.24
cT1	98	80	9	20	51	1 [Reference]	NA	1 [Reference]	NA
Gleason score									
8-10	33	29	10	8	11	1.83 (1.22-2.74)	.003	1.30 (0.84-2.00)	.23
6-7	173	132	20	31	81	1 [Reference]	NA	1 [Reference]	NA
Interaction of comorbidity by ADT	206	161	30	39	92	2.92 (1.46-5.84)	.002	3.46 (1.69-7.07)	.001
Comorbidity subgroup									
Moderate/severe	49	46	4	21	21	1.50 (0.93-2.42)	.10	1.55 (0.95-2.53)	.08
None/minimal	157	115	26	18	71	1 [Reference]	NA	1 [Reference]	NA
Treatment for moderate/severe comorbidity subgroup									
EBRT plus 6 mo ADT	24	23	1	15	7	1.97 (1.10-3.54)	.02	2.83 (1.51-5.28)	.001
EBRT	25	23	3	6	14	1 [Reference]	NA	1 [Reference]	NA
Treatment for none/minimal comorbidity subgroup									
EBRT plus 6 mo ADT	78	54	5	7	42	0.68 (0.47-0.97)	.04	0.82 (0.54-1.23)	.33
EBRT	79	61	21	11	29	1 [Reference]	NA	1 [Reference]	NA
Time-dependent PSA failure variable	108	85	30	15	40	1.50 (1.08-2.08)	.01	1.51 (1.03-2.23)	.04

Abbreviations: ADT, androgen deprivation therapy; AHR, adjusted hazard ratio; AJCC, American Joint Committee on Cancer; CVD, cardiovascular disease; DFCI, Dana-Farber Cancer Institute; EBRT, external beam radiotherapy; HR, hazard ratio; NA, not applicable; PSA, prostate-specific antigen.

^a^ SI conversion factor: To convert PSA to μg/L, multiply by 1.0.

For EORTC 22961 (Table 4), significant factors associated with ACM from the univariable model included older age (HR, 1.06 [95% CI, 1.04-1.08] per year; *P* < .001), higher baseline PSA (HR, 1.28 [95% CI, 1.09-1.50] per ng/mL; *P* = .003), cT3 to cT4 category (HR, 1.44 [95% CI, 1.01-2.04]; *P* = .04), Gleason score of 8 to 10 (HR, 1.69 [95% CI, 1.25-2.28]; *P* < .001), performance status of 1 to 2 (HR 1.63 [95% CI, 1.07-2.47]; *P* = .02), EBRT plus 36 months of ADT for the subgroup with a performance status of 0 (HR, 0.66 [95% CI, 0.49-0.89]; *P* = .007), and PSA failure (HR, 3.79 [95% CI, 2.87-5.02]; *P* < .001). External beam radiotherapy plus 36 months of ADT for the subgroup with a performance status of 1 to 2 (HR, 0.97 [95% CI, 0.55-1.69]; *P* = .90) was not significant, although only 148 men in the entire cohort (15.3%) had a performance status of 1 to 2. In the multivariable model, significant factors associated with ACM included older age (AHR, 1.07 [95% CI, 1.05-1.10] per year; *P* < .001), Gleason score of 8 to 10 (AHR, 1.42 [95% CI, 1.05-1.93]; *P* = .02), performance status of 1 to 2 (AHR, 1.65 [95% CI, 1.08-2.52]; *P* = .02), and PSA failure (AHR, 3.98 [95% CI, 2.92-5.44]; *P* < .001). Of note, there were no significant interactions between comorbidity and treatment arm in the univariable (*P* = .25) and multivariable (*P* = .24) models. As a sensitivity analysis, PSA failure using the prospective definition was also significantly associated with ACM (AHR, 5.42 [95% CI, 3.96-7.43]; *P* < .001) (see eTable 1 in Supplement 1 for cross-tabulation between the PSA failure definitions and eTable 2 in Supplement 1 for regression results).

**Table 4.** Univariable and Multivariable Cox Proportional Hazards Regression Models for All-Cause Mortality for the EORTC 22961 Trial Using the PSA (Nadir Plus 2) Definition

Variable	No. of men	No. of deaths by cause				Univariable analysis		Multivariable analysis with PSA failure (nadir plus 2)	
		All	Prostate cancer	CVD	Other	HR (95% CI)	*P* value	AHR (95% CI)	*P* value
Age, y	967	230	75	56	99	1.06 (1.04-1.08)	<.001	1.07 (1.05-1.10)	<.001
Logarithm of PSA level, ng/mL^a^	967	230	75	56	99	1.28 (1.09-1.50)	.003	1.14 (0.97-1.35)	.12
AJCC tumor category									
cT3-cT4	754	193	65	45	83	1.44 (1.01-2.04)	.04	1.39 (0.96-2.00)	.08
cT1-cT2	213	37	10	11	16	1 [Reference]	NA	1 [Reference]	NA
AJCC nodal category									
cN1	82	19	13	1	5	0.98 (0.61-1.57)	.93	1.09 (0.66-1.81)	.74
cN0	885	211	62	55	94	1 [Reference]	NA	1 [Reference]	NA
Gleason score									
8-10	185	60	24	17	19	1.69 (1.25-2.28)	<.001	1.42 (1.05-1.93)	.02
6-7	742	154	47	36	71	1 [Reference]	NA	1 [Reference]	NA
Unknown	40	16	4	3	9	1.73 (1.03-2.90)	.04	1.29 (0.76-2.19)	.34
Interaction of performance status by ADT	967	230	75	56	99	1.45 (0.77-2.74)	.25	1.47 (0.78-2.77)	.24
Performance status subgroup									
1-2	148	50	11	16	23	1.63 (1.07-2.47)	.02	1.65 (1.08-2.52)	.02
0	819	180	64	40	76	1 [Reference]	NA	1 [Reference]	NA
Performance status 1-2 subgroup by treatment									
EBRT plus 36 mo ADT	77	22	3	9	10	0.97 (0.55-1.69)	.90	1.47 (0.83-2.61)	.19
EBRT plus 6 mo ADT	71	28	8	7	13	1 [Reference]	NA	1 [Reference]	NA
Performance status 0 subgroup by treatment									
EBRT plus 36 mo ADT	414	76	25	16	35	0.66 (0.49-0.89)	.007	1.00 (0.73-1.37)	.99
EBRT plus 6 mo ADT	405	104	39	24	41	1 [Reference]	NA	1 [Reference]	NA
Time-dependent PSA failure variable (nadir plus 2)	290	89	64	10	15	3.79 (2.87-5.02)	<.001	3.98 (2.92-5.44)	<.001

Abbreviations: ADT, androgen deprivation therapy; AHR, adjusted hazard ratio; AJCC, American Joint Committee on Cancer; CVD, cardiovascular disease; EBRT, external beam radiotherapy; EORTC, European Organization for Research and Treatment of Cancer; HR, hazard ratio; NA, not applicable; PSA, prostate-specific antigen.

^a^ SI conversion factor: To convert PSA to μg/L, multiply by 1.0.

The Figure shows the evolution of the AHR values of PSA failure by landmark time points. For DFCI 95-096, a numerically greater AHR of 3.06 (95% CI, 1.20-7.81) was noted at year 1. Subsequently, AHR values ranged from 1.29 (95% CI, 0.83-2.01) to 1.61 (95% CI, 1.05-2.49) from years 2 to 6. For EORTC 22961, a greater AHR of 7.56 (95% CI, 3.76-15.17) was observed at year 1. Subsequently, AHR values ranged from 3.20 (95% CI, 1.90-5.38) to 4.88 (95% CI, 3.25-7.33) from years 2 to 6. In both trials, wide 95% CIs were noted at year 1, owing to fewer men with PSA failure.

**Figure.**
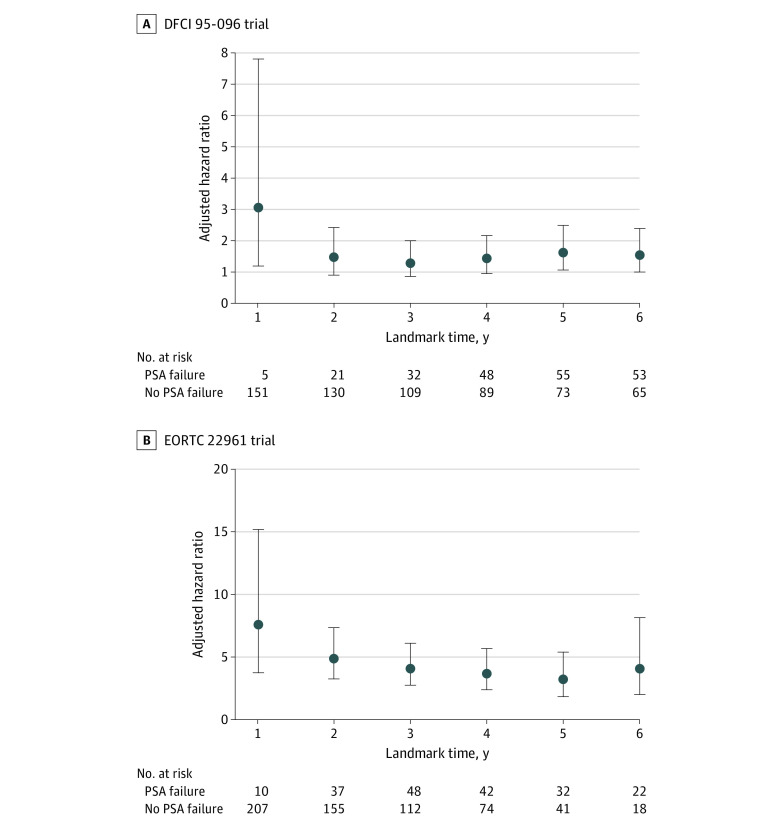
Plots of Adjusted Hazard Ratios of Increased Levels of Prostrate-Specific Antigen After Treatment (PSA Failure) by Landmark Times DFCI indicates Dana-Farber Cancer Institute; EORTC, European Organisation for Research and Treatment of Cancer.

## Discussion

In this analysis of the DFCI 95-096 and EORTC 22961 randomized clinical trials, we report that PSA failure may be associated with a higher risk of death in locally advanced vs localized prostate cancer. For the DFCI 95-096 trial, which included men with localized disease, PSA failure was associated with a lower risk of ACM (AHR, 1.51 [95% CI, 1.03-2.23]; *P* = .04). For the EORTC 22961 trial, which included men with locally advanced disease, PSA failure was associated with a higher risk of ACM (AHR, 3.98 [95% CI, 2.92-5.44]; *P* < .001). These results suggest that PSA failure may be associated with a higher risk of death in locally advanced compared with localized prostate cancer.

Men with locally advanced prostate cancer who experience PSA failure may be at a greater risk of death because they may harbor more aggressive prostate cancer at the time of biochemical recurrence. Analyses from randomized clinical trials have reported that PSA doubling time after EBRT is a strong prognostic factor for ACM after completion of EBRT.^13,14^ The median PSA doubling time for those who experienced PSA failure in the EORTC 22961 trial was 5.0 months, shorter than the 13.0 months reported in the DFCI 95-096 trial. Prostate-specific antigen doubling time may therefore be a critical factor driving the increased risk of death for locally advanced prostate cancer.

This analysis supports the notion that PSA failure may have a different prognostic meaning for different patient populations. It builds off the previous analysis of Giacalone et al,^7^ which showed that PSA failure was significantly associated with ACM in men with no/minimal comorbidities vs those with moderate/severe comorbidities. We now extend this concept for locally advanced vs localized disease.

Interestingly, the ICECaP working group also reported an improved patient-level correlation between PSA event–free and overall survival (Kendall τ = 0.56) in the subgroup of men who underwent at least 2 years of ADT.^8^ This subgroup included men from the EORTC 22961^10^ as well as the DART 01/05 trial,^3^ Radiation Therapy Oncology Group (RTOG) 92-02 trial,^15^ and a French study.^16^ On the other hand, correlations between PSA event–free and overall survival were much weaker for the entire population (0.43) as well as the subgroup of men with high-risk disease (0.44).

This study may have clinical implications for men who experience PSA failure after treatment for locally advanced disease. It may prompt clinicians to order prostate-specific positron emission tomography scans, which can detect metastatic disease at much lower PSA values than conventional computed tomography or bone scans.^17^ Men with suspected metastatic disease may be eligible for early initiation of salvage ADT as well as novel secondary antiandrogens, abiraterone acetate, or docetaxel, based on recent randomized clinical trials reporting a survival benefit in the de novo metastatic setting.^18,19,20,21^ Such men may also be candidates for metastasis-directed therapy.^22,23^ For men with nonmetastatic disease, early salvage ADT alone could be considered.^24^ Furthermore, trials evaluating treatment intensification with novel antiandrogens in addition to ADT at the time of PSA failure could be considered.^25^

This study also supports strategies for minimizing the risk of PSA failure in men with aggressive prostate cancer during upfront definitive ADT and EBRT. Brachytherapy boost could be considered for locally advanced disease to minimize the risk of PSA^5^ and local failure.^26,27^ A recently activated trial^28^ is now randomizing men with high-risk prostate cancer and persistently detectable PSA levels after EBRT and 6 to 12 months of ADT^29^ to continued ADT with or without abiraterone/prednisone and apalutamide. Metastasis-free survival is the primary end point.

### Strengths and Limitations

A key strength of this study is the analysis of randomized data. This allowed for the evaluation of PSA failure with respect to treatment effect. In both trials, the effect of treatment for men with minimal competing risk (no/minimal comorbidities for DFCI 95-096 or a performance score of 0 for EORTC 22961) was no longer significant when PSA failure was included in the models. Furthermore, we were able to conduct a landmark analysis to evaluate whether earlier PSA failure may be associated with ACM. Men with early PSA failure (at year 1 for DFCI 95-096 and EORTC 22961) may have a higher risk of ACM, although the 95% CIs were wide owing to the lower numbers of patients at risk.

This study has some limitations. An important weakness is that it was based on only 2 mature randomized clinical trials. In addition, the DFCI 95-096 sample size was rather small. Multiple randomized clinical trials are needed to validate whether PSA failure holds greater prognostic potential for more aggressive prostate cancer. Sufficiently powered studies could also evaluate whether PSA failure has differing prognostic potential based on the duration of ADT previously received. In addition, PSA failure per the nadir plus 2 definition was calculated retrospectively for the EORTC 22961 trial. However, in our sensitivity analysis, PSA failure calculated per the prospective definition was also significantly associated with ACM. Furthermore, PSA doubling times may have been less accurate, because very low PSA values from the time of PSA nadir rather than the time of PSA recurrence to the time of salvage treatment were used. However, this was necessary to obtain a PSA doubling time for all patients. It is also unclear whether the association with PSA failure is valid for men who have tumors with neuroendocrine features that produce low levels of PSA.^30^ Of note, the RTOG 0521 trial, which enrolled 52.8% of patients with Gleason scores of 9 to 10, reported that the addition of docetaxel to ADT and EBRT improved overall survival without a significant improvement in PSA relapse–free survival.^31^

## Conclusions

The findings of this cohort study suggest that increased PSA levels after treatment may be associated with worse outcomes for men with locally advanced vs localized prostate cancer. However, these results need to be validated with data from additional randomized clinical trials involving localized and locally advanced prostate cancer.

## Notes

Supplement 1. **eTable 1.** Cross-tabulation of PSA Failure by the Nadir Plus 2 Definition vs the Prospective Definition for EORTC 22961 **eTable 2.** Univariable and Multivariable Cox Proportional Hazards Regression Models for All-Cause Mortality for EORTC 22961 Using the Prospective PSA Failure Definition **eFigure 1.** CONSORT Diagram for DFCI 95-096 **eFigure 2.** CONSORT Diagram for EORTC 22961

Supplement 2. DFCI 95-096 Trial Protocol

Supplement 3. EORTC 22961 Trial Protocol
