# Supplementary material for: Association of Increased Prostate-Specific Antigen Levels After Treatment and Mortality in Men With Locally Advanced vs Localized Prostate Cancer: A Secondary Analysis of 2 Randomized Clinical Trials
Source: JAMA Netw Open. 2021 May 17;4(5):e2111092. doi: 10.1001/jamanetworkopen.2021.11092 (PMC8129819; doi:10.1001/jamanetworkopen.2021.11092)
Supplement: Supplement 1. — eTable 1. Cross-tabulation of PSA Failure by the Nadir Plus 2 Definition vs the Prospective Definition for EORTC 22961 eTable 2. Univariable and Multivariable Cox Proportional Hazards Regression Models for All-Cause Mortality for EORTC 22961 Using the Prospective PSA Failure Definition eFigure 1. CONSORT Diagram for DFCI 95-096 eFigure 2. CONSORT Diagram for EORTC 22961 [file jamanetwopen-e2111092-s001.pdf]

Supplementary Online Content

King MT, Chen MH, Collette L, Neven A, Bolla M, D'Amico AV. Association of increased prostate-specific antigen levels after treatment and mortality in men with locally advanced vs localized prostate cancer: a secondary analysis of 2 randomized clinical trials. *JAMA Netw Open*. 2021;4(5):e2111092. doi:10.1001/jamanetworkopen.2021.11092

eTable 1. Cross-tabulation of PSA Failure by the Nadir Plus 2 Definition vs the Prospective Definition for EORTC 22961

eTable 2. Univariable and Multivariable Cox Proportional Hazards Regression Models for All-Cause Mortality for EORTC 22961 Using the Prospective PSA Failure Definition

eFigure 1. CONSORT Diagram for DFCI 95-096

eFigure 2. CONSORT Diagram for EORTC 22961

This supplementary material has been provided by the authors to give readers additional information about their work.

© 2021 King MT et al. *JAMA Network Open*.

eTable 1. Cross-tabulation of PSA Failure by the Nadir Plus 2 Definition vs the Prospective Definition for EORTC 22961

	No PSA failure (prospective)	PSA failure (prospective)
No PSA failure (nadir + 2)	673	4
PSA failure (nadir + 2)	39	251

© 2021 King MT et al. *JAMA Network Open*.

eTable 2. Univariable and Multivariable Cox Proportional Hazards Regression Models for All-Cause Mortality for EORTC 22961 Using the Prospective PSA Failure Definition

Abbreviations: ADT – androgen deprivation therapy; AHR – adjusted hazard ratio; AJCC – American Joint Committee on Cancer; CV – cardiovascular; HR – hazard ratio; OC – other causes; PC – prostate cancer; PS – performance status; PSA – prostate-specific antigen; RT – radiation therapy; (t) – time-dependent variable.

			Univariable analysis		Multivariable analysis with PSA failure (prospective)	
	No. Men	No. Deaths (PC, CV, OC)	HR	p-value	AHR	p-value
Age, years	967	230 (75, 56, 99)	1.06 [1.04, 1.08]	<0.001	1.07 [1.05, 1.10]	<0.001
Logarithm of PSA level, ng/mL	967	230 (75, 56, 99)	1.28 [1.09, 1.50]	0.003	1.10 [0.93, 1.30]	0.26
AJCC tumor category						
T3-T4	754	193 (65, 45, 83)	1.44 [1.01, 2.04]	0.04	1.38 [0.96, 1.99]	0.08
T1-T2	213	37 (10, 11, 16)	1.00 (reference)	-	1.00 (reference)	-
AJCC nodal category						
N1	82	19 (13, 1, 5)	0.98 [0.61, 1.57]	0.93	1.12 [0.67, 1.85]	0.67
N0	885	211 (62, 55, 94)	1.00 (reference)	-	1.00 (reference)	-
Gleason score						
Unknown	40	16 (4, 3, 9)	1.73 [1.03, 2.90]	0.04	1.35 [0.80, 2.29]	0.26
8-10	185	60 (24, 17, 19)	1.69 [1.25, 2.28]	<0.001	1.38 [1.02, 1.87]	0.04
6-7	742	154 (47, 36, 71)	1.00 (reference)	-	1.00 (reference)	-
Interaction	967	230 (75, 56, 99)	1.45 [1.07, 2.47]	0.25	1.66 [0.88, 3.15]	0.12
PS						
1-2	148	50 (11, 16, 23)	1.71 [1.12, 2.61]	0.01	1.64 [1.07, 2.50]	0.02
0	819	180 (64, 40, 76)	1.00 (reference)	-	1.00 (reference)	-
PS1-2: treatment arm						
RT + 36 month ADT	77	22 (3, 9, 10)	0.97 [0.55, 1.69]	0.90	1.70 [0.95, 3.03]	0.07
RT + 6 month ADT	71	28 (8, 7, 13)	1.00 (reference)		1.00 (reference)	-
PS0: treatment arm						
RT + 36 month ADT	414	76 (25, 16, 35)	0.66 [0.49, 0.89]	0.007	1.02 [0.75, 1.39]	0.90
RT + 6 month ADT	405	104 (39, 24, 41)	1.00 (reference)	-	1.00 (reference)	-
PSA failure (t)	255	91 (70, 8, 13)	4.80 [3.63, 6.35]	<0.001	5.42 [3.96, 7.43]	<0.001

© 2021 King MT et al. *JAMA Network Open*.

eFigure 1. CONSORT Diagram for DFCI 95-096.

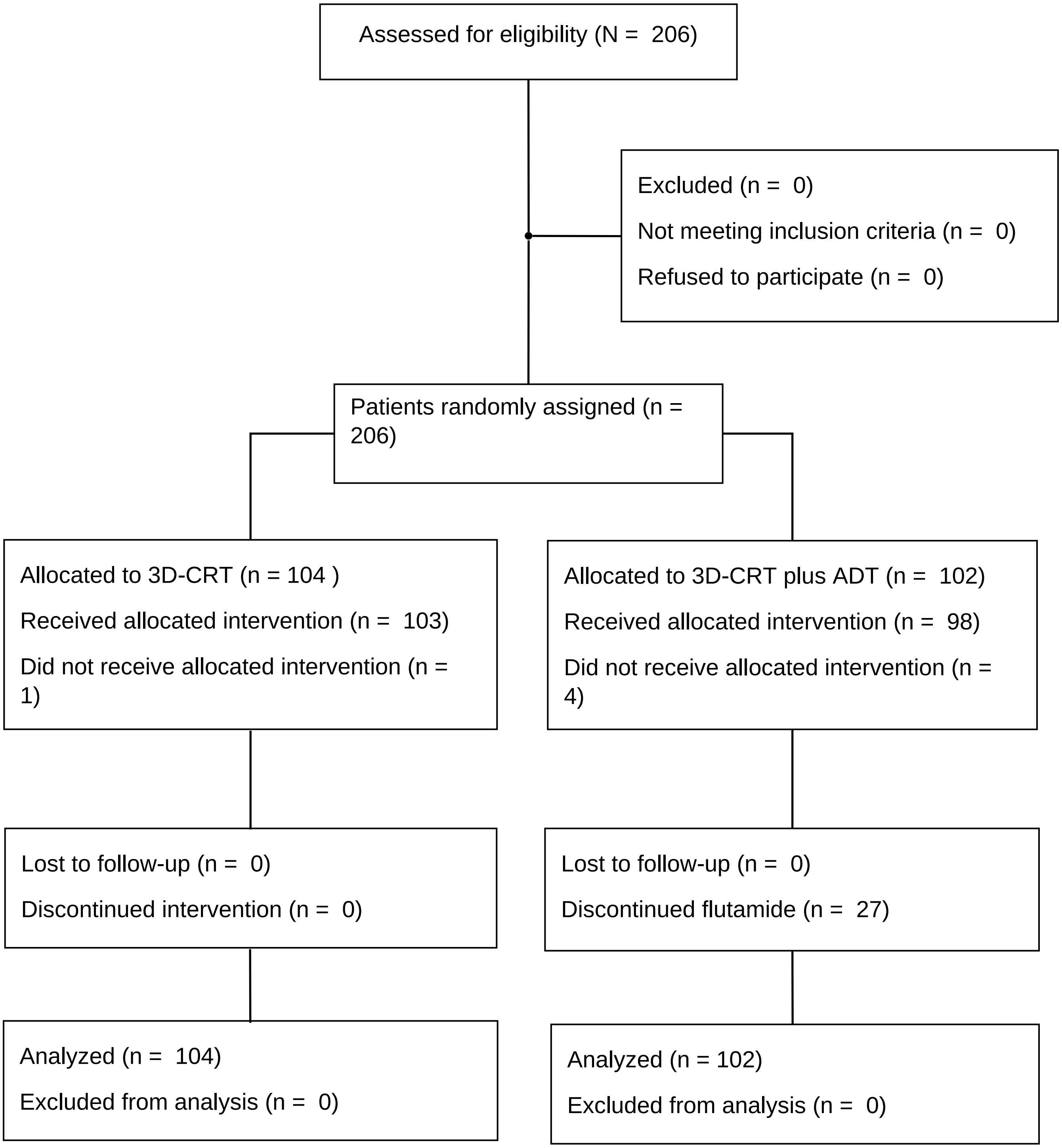

© 2021 King MT et al. *JAMA Network Open*.

eFigure 2. CONSORT Diagram for EORTC 22961

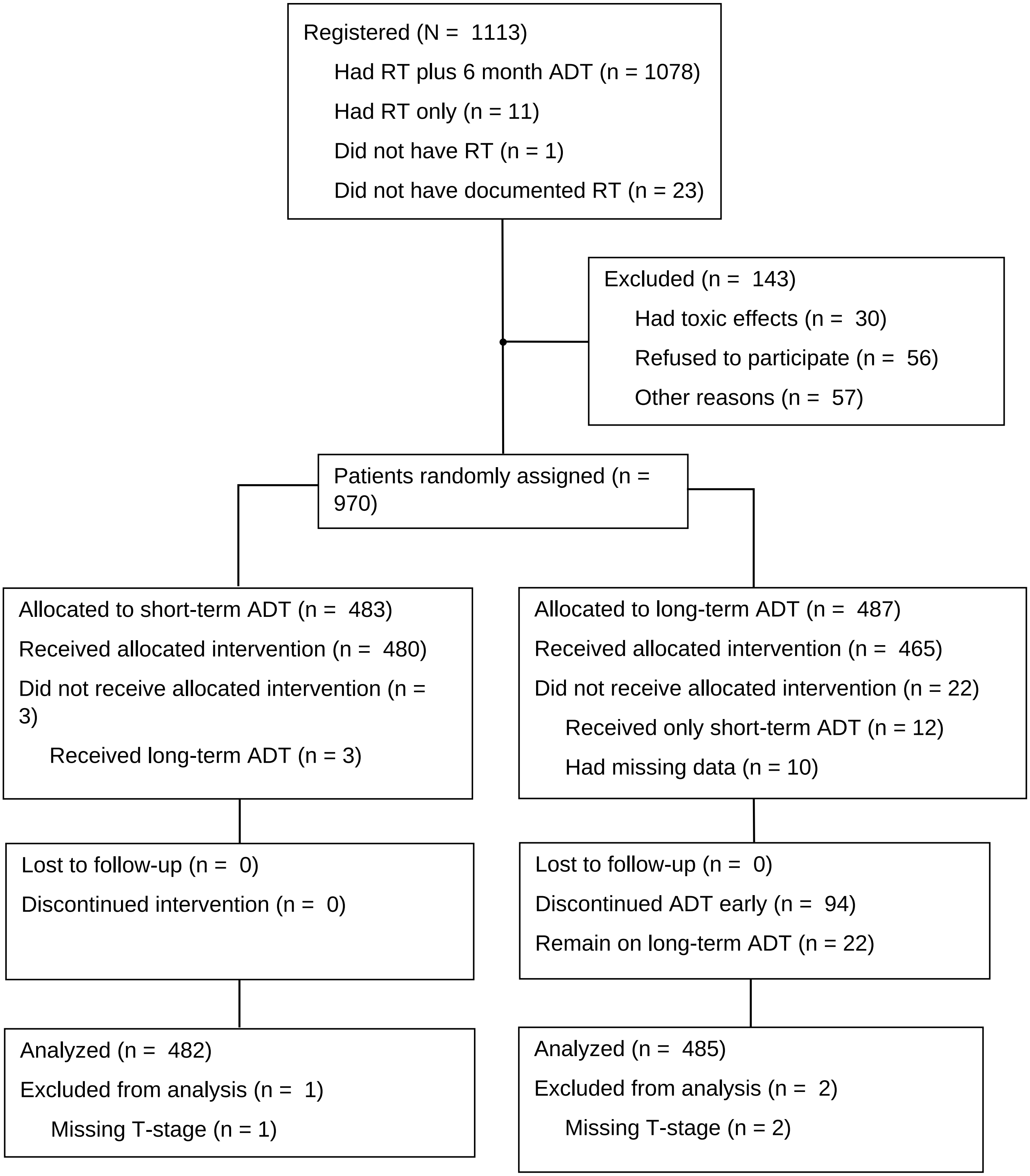

© 2021 King MT et al. *JAMA Network Open*.
