# Supplementary material for: Association of Increased Prostate-Specific Antigen Levels After Treatment and Mortality in Men With Locally Advanced vs Localized Prostate Cancer: A Secondary Analysis of 2 Randomized Clinical Trials
Source: JAMA Netw Open. 2021 May 17;4(5):e2111092. doi: 10.1001/jamanetworkopen.2021.11092 (PMC8129819; doi:10.1001/jamanetworkopen.2021.11092)
Supplement: Supplement 2. — DFCI 95-096 Trial Protocol [file jamanetwopen-e2111092-s002.pdf]

STATUS PAGE

PROTOCOL **95-096**

Closed to New Accrual

Closure Effective Date: 04/03/01

No new subjects may be enrolled in the study as described above. Any questions regarding this closure should be directed to the study's Principal Investigator

DANA-FARBER CANCER INSTITUTE

DANA-FARBER / HARVARD CANCER CENTER

Office for Human Research Studies

Protocol Front Sheet

DFCI Protocol No.: 95-096

1. PROTOCOL TITLE AND VERSION

Title: A Phase III Trial of External Beam of Radiotherapy +/- Total Androgen Suppression for High-Risk Clinically Organ Confined Prostate Cancer

Protocol Version No./ Date: December 14, 1999 **Sponsor Study Number:** N/A

2. DF/HCC STUDY CONTACT INFORMATION

Study Contact for Questions: Marian J. Loffredo, RN **Email:** mloffredo@lroc.harvard.edu **Phone:** 617-355-7264

OVERALL AND SITE RESPONSIBLE INVESTIGATORS (List only those under DFCI IRB, *i.e.*, from institutions listed in Section 6 below)

**Overall PI:**	Anthony V. D'Amico, M.D. Ph.D.	**Phone:**	617-632-6328	**Institution(s):**	BWH/DFCI
**Site Responsible PI:**	Philip Kantoff, M.D.	**Phone:**	617-632-1914	**Institution(s):**	DFCI

Co-Investigators name (institution)**:** None

Additional Study Staff/Contacts name & institution (no more than one person per institution listed in section 6 below)**:** Marian J. Loffredo, RN BWH/DFCI

3. DRUG / DEVICE INFORMATION N/A:

Drugs, Biologics, Devices (name & IND/IDE#): N/A

IND/IDE held by: (Check if IND/IDE exempt: ☒)

Investigational Drug Brochure (IDB) Version No./ Date: N/A

(Check if already on file with OHRS:☐)

4. PROTOCOL COORDINATION, FUNDING, PHASE, MODE, TYPE ETC.

Protocol Coordinated By:
DF/HCC Investigator

Cancer Related: Yes If yes:
Primary Disease Program:
Prostate Cancer
or
Primary Discipline Based Program:
Radiation Oncology

Funding/Support (check all that apply)**:**
☐ Industry:
☐ Federal Organization:
Grant #:
☐ Internal Funding:
☐ Non-Federal:
☐ Other:

Phase: Phase 3

Multi-Center (i.e., non-DF/HCC site participation)**:**
Yes

Protocol Type: Therapeutic
If Ancillary, provide parent protocol #:

Protocol Involves (check all that apply as listed in the protocol document, even if not part of the research but is mandated by the protocol document)**:**

☐ Chemotherapy
☐ Immunotherapy
☐ Surgery
☐ Bone Marrow/Stem Cell Transplant
☐ Cell Based Therapy
☐ Gene Transfer (use of recombinant DNA)
☒ Radiation Therapy
☒ Hormone Therapy
☐ Vaccine
☐ Data Repository
☐ Exercise/Physical Therapy
☐ Genetic Studies
☐ Human Material Banking
☐ Human Material Collection
☐ Medical Record Review
☐ Questionnaires/Surveys/Interviews
☒ Radiological Exams
☐ Required Biopsy Study
☐ Human Embryonic Stem Cell
☐ Other:

5. SUBJECT POPULATION (also applies to medical record review and specimen collection studies)

Total Study-Wide Enrollment Goal: 270 - study stopped at 206 **Greater than 25% of the overall study accrual will be at DF/HCC:** ☐ Yes ☒ No

Total DF/HCC Estimated Enrollment Goal: N/A **Adult Age Range:** >30 **Pediatric Age Range:** 0

Will all subjects be recruited from pediatric clinics? ☐ Yes ☒ No

If enrolling both adults and pediatric subjects, anticipated percent of pediatric subjects: none

6. INSTITUTIONAL PARTICIPANTS UNDER DFCI IRB

Dana-Farber/Harvard Cancer Center: (check all that apply)
☐ Beth Israel Deaconess Medical Center
☒ Brigham and Women's Hospital
☐ Children's Hospital Boston
☒ Dana-Farber Cancer Institute
☐ Dana-Farber Cancer Institute at Faulkner Hospital
☐ Dana-Farber Cancer Institute at Londonderry Hospital
☐ Dana-Farber Cancer Institute at Milford Hospital
☐ Dana-Farber Cancer Institute at South Shore
☐ Massachusetts General Hospital
☐ Massachusetts General Hospital/North Shore Cancer Center
☐ Massachusetts General Hospital Radiation Oncology at Emerson Hospital

DF/PCC Network Affiliates: (check all that apply)
☐ Cape Cod Healthcare (Hyannis, MA; Mashpee, MA)
☐ Lowell General Hospital (Lowell, MA)
☐ New Hampshire Oncology-Hematology-P.A. (Concord, NH; Hooksett, NH; Laconia, NH)
☐ Newton-Wellesley Hospital (Newton, MA)

7. DF/HCC INITIATED STUDIES ONLY - INSTITUTIONAL PARTICIPANTS UNDER OTHER IRB (N/A:)

DF/HCC Multi-Center Protocols: (list institution/location)

JCRT - Metro West Medical Center	Framingham, MA
JCRT - BIDMC	Waltham
JCRT - South Surburban	South Weymouth
(JCRT - formerly under DFCI	

DF/PCC Network Affiliates: (list institution/location)

Saint Anne's Hospital	Fall River, MA

until disbanded around 1998.
All subjects only in long-term follow-up for survival at these former JCRT sites.

8. OTHER INVOLVED SITES NOT UNDER DFCI IRB (N/A:)

Please list other sites that will be involved in portions of the study, for example, ocular screening and monitoring that will be conducted at Joslin Diabetes Center: (list institution/location)

Protocol Number: 95-096

Approval Date: 9/21/95 (HPC meeting date when protocol/consent approved)

Activation Date: 9/29/95 (Date when protocol open to patient entry)

Approval signatures are on file in the Office for Human Research Studies, tel. 617-632-3029.

Date Posted	Revised Sections	IRB/HPC Approval Date	OHRS Version Date
11/29/95	Front Sheet: Investigator Names added		-
2/12/96	Sections 3.14 and 3.18 Consent Form		-
2/14/96	Sections: 3.12, 3.14, 5.11, 6.1, and 6.2		-
2/27/96	Front Sheet, Investigator and Part. Institution added		-
5/9/96	Schema and Section 3.18		-
5/16/96	Sections 3.1 and 6.0 Protocol repagination due to revision		-
9/18/96	Section 3.14 Protocol repagination due to revision		-
10/24/96	Section 3.18 and page 11		-
9/26/96	Consent Form due to HPC review		-
1/9/97	Sections: 3.1, 4.0, 5.3, 6.0, Consent Form, Appendix d, Sections 2.2, 3.100, 5.11, 5.13, 5.14, 5.16, 5.17, 5.18, 5.21, 5.31, 5.411 (lines 1 and 2) 5.423 (line 4), 5.4245, 6.1, (footnote 2, 6) 6.1 (footnote 5) 6.2, 6.21, 6.2,, 7.1, 7.21, 7.211, 7.213, 7.215, 7.221, 7.23, 7.24, 7.31, 9.2 (lines 1 and 2) 9.3, 9.4		-
8/14/97	Consent Form		-
3/3/98	Consent Form, Protocol Cover Sheet, Schema, Section 3.103, 3.108, 3.22, 4.3, 4.5		-
4/2/98	Section 5.424 Page 8 line 5		-
4/22/98	Section 8.1-8.3	6/3/98	-
8/11/98	Section 5.424, Consent Form, 6.0, 6.2 Sections 8.1-8.3	9/16/98	-
12/16/98	Section 5.0 Treatment Plans (page 5) replaced, Consent Form	1/20/99	-
7/22/99	Protocol Replaced: Schema & Section 5.22	8/25/99	-
12/14/99	Protocol – Section 3.22	1/12/00	-
08/06/07	On Hold Alert Page posted due to Continuing Review pending; All research must stop	N/A	-
10/02/07	Correction: front sheet replaced	N/A	-
12/13/07	Study renewal due to Continuing Review #9; Hold alert page removed	12/12/07	-
10/27/08	Study renewal (footer replaced) due to Continuing Review #10	10/24/08	-
10/23/09	Study renewal due to Continuing Review #11	10/17/09	-
10/13/10	Front Sheet replaced and Study renewal due to Continuing Review #12	10/10/10	-
11/05/10	BIDMC site ON HOLD due BIDMC Strategic Hold OE #16	N/A	-
05/17/11	Correction: removing administrative hold at BIDMC because BIDMC is not a participating site	N/A	-
10/06/11	Study renewal due to Continuing Review #13	10/01/11	-
09/28/12	Study renewal/Consent Form footer replaced due to Continuing Review #14	09/26/12	N/A
09/26/13	ON HOLD: All research must stop due to lapsed Continuing Review. Study approval expired.	N/A	N/A

09/30/13	On HOLD removed: Study renewal / Consent Form footer replaced due to Continuing Review #15	09/26/13	N/A
09/26/14	ON HOLD: All research must stop due to lapsed Continuing Review. Study approval expired 09/26/14.	N/A	N/A
09/26/14	On HOLD removed: Study renewal / Consent Form footer replaced due to Continuing Review #16	09/19/14	N/A
**Date Posted**	**Revised Sections**	**IRB Approval Date**	**OnCore Version Date**
09/14/15	Study renewal/Consent Form footer replaced due to Continuing Review #17	07/30/15	07/31/15
07/13/16	Study renewal/ Consent Form footer replaced due to Continuing Review #18	06/27/16	07/12/16
**Date Posted**	**Revised Sections**	**Approved Date**	**Version Date (OnCore)**
06/21/17	Study renewal/ Consent Form footer replaced due to Continuing Review #19	06/05/17	06/19/17
04/12/2018	Study renewal/Consent Form footer replaced per Continuing Review #20	04/09/2018	04/11/2018
04/08/19	Study renewal/Consent Form footer replaced due to Continuing Review #21	04/08/19	04/08/19

Revised August 11, 1998

Table of Contents

	Page
Protocol Schema	1
1.0 Introduction	2
2.0 Study Objectives	3
3.0 Patient Selection	3
4.0 Patient Entry	4
5.0 Treatment Plan	5
6.0 Required Data	10
7.0 Modality Review	12
8.0 Statistics	14
9.0 Central Pathology Review	15
10.0 Race/Ethnicity	15
References	17
Appendices	

Revised July 22, 1999

SCHEMA

Patient Eligibility

Clinically Organ Confined Prostate Cancer ($T_{1b,1c,}T_{2a,2b}$)
Bone Scan (-)**
(-) LN assessment (Lap, CT, or MR)

PSA > 20 ng/ml and PSA ≤ 40 ng/ml
or
Biopsy Gleason score ≥ 7 (Maximum PSA = 40 ng/ml)
or
PSA > 10 ng/ml, PSA ≤ 20 ng/ml and biopsy Gleason score 2 - 6
or
Endorectal coil MR (+) ECE or SVI and PSA > 4 ng/ml and PSA ≤ 10 ng/ml and biopsy Gleason score 5 - 6

RANDOMIZE

2 months of External Beam Radiation Therapy*

2 months of Total Androgen Suppression#

2 months of Total Androgen Suppression# plus External Beam Radiation Therapy*

2 months of Total Androgen Suppression#

Post Therapy Evaluation

PSA, Digital Rectal Exam every 3 months

*** Recommended External Beam Radiation Therapy 45 to 50.4 Gy (25-28 doses of 1.8 Gy) delivered to prostate + seminal vesicles (up to a) 2 cm margin, a boost for an additional 16 to 22 Gy (8-11 doses of 2.0 Gy) to prostate + (up to a) 2 cm margin.**
# Total Androgen Suppression: Leuprolide acetate (7.5 mg IM q month or 22.5 mg. IM q 12 weeks) and Flutamide 250 mg po q 8° or Zoladex (3.6 mg SC q month or 10.8 mg. q 12 weeks into the abdominal wall) and Flutamide 250mg po q 8°, 2 months prior to, 2 months concurrent with, and 2 months after radiation therapy
****Bone scan is unnecessary in patients with a PSA <10 ng/ml, and a Gleason Score of ≤ 3+4=7**

1.0 Introduction

Rationale

A retrospective multivariate analysis examining the predictive value of prostate specific antigen (PSA), biopsy Gleason sum, endorectal coil magnetic resonance imaging (MR) findings and clinical stage on postoperative PSA failure was performed at the University of Pennsylvania[1]. The p-values from this analysis are shown in Table 1. The results of the multivariate analysis provided the basis on which subgroups at high, intermediate, and low risk for early (2 year) postoperative biochemical failure could be determined. These subgroups are defined in terms of the independent prognostic indicators from the multivariate analysis, namely PSA and biopsy Gleason sum as shown in Table 2. Realizing that both the endorectal coil MR finding of seminal vesicle invasion (SVI) and extracapsular extension (ECE) also independently predicted for postoperative PSA failure on multivariate analysis provided the ability to further subdivide the intermediate risk patients further into high and low risk as shown in Figures 1 - 4.

A second independently generated surgical data set from Johns Hopkins University[2] confirmed the risk stratification proposed from the multivariate analysis of the Univ. of Pennsylvania data set as shown in Table 3. Specifically, the incidence of extraprostatic disease in the low, intermediate, and high risk subgroups as defined in Table 2 from the PENN data set correspond to <46%, 27% - 81%, > 52% respectively in the Hopkin's data set. Therefore, the Hopkins experience confirms the risk stratification proposed.

Conflicting data[4-5] exists as to whether 3 - 4 months of total androgen suppression pathologically downstages patients when given prior to radical prostatectomy in clinical stage T2c and T3 patients. This may be explained by data from Sullivan[6] which concludes that in this patient population only 27% and 61% of patients have achieved undetectable PSA levels after 3 and 4 months of total androgen suppression (TAS) respectively. However, using 6 - 7 months of TAS, 94 - 100% of patients achieved undetectable PSA levels.

In patients with bulky stage T2b - T4 tumors, recent data from RTOG 86-10[7] shows an increase in the 5 year actuarial freedom from local (54% versus 29%, $p < .001$) and biochemical failure (36% versus 15%, $p < .001$) for patients treated with 4 months of TAS and radiation therapy versus radiation therapy alone. Distant disease free survival (66% versus 59%; $p = .09$) also approached statistical significance.

Hypothesis

The use of 6 months of TAS in combination with local field radiation therapy for patients with early stage (T1b - T2b) disease may improve overall survival by improving both local and distant control in a population of patients known to have high 2 year actuarial postoperative PSA failure rates.

Recent data from University of California San Francisco[3] on 57 clinically localized prostate cancer patients found that while both endorectal coil MR and the number of positive biopsies were superior to transrectal ultrasound ($p = .01$, .02 respectively) and the primary Gleason pattern ($p = .06$, .06 respectively) using an ROC analysis, neither was superior to the other ($p = .12$) in the prediction of extracapsular extension. Numbers, however, were too small to make a definitive statement regarding the relative efficacy of endorectal coil MR versus the number of positive pretreatment biopsies on ultimate outcome (i.e. freedom from PSA failure and overall survival).

Using the transrectal ultrasound biopsy data, the predictive value of the number of positive biopsies obtained during the standard sextant biopsy can be directly compared to clinical stage, PSA, biopsy Gleason sum, and the endorectal coil MRI findings for the outcome of post-therapy PSA failure using a multivariable analysis.

2.0 Study Objectives

2.1 To determine if the 2 year freedom from PSA failure is increased in patients receiving total androgen suppression (TAS) and radiation therapy (RT) compared to those patients receiving radiation therapy alone in a population of patients known to be at high risk for early PSA failure after radical prostatectomy.

2.2 To evaluate the quality of life of patients receiving local field RT + TAS using DFCI protocol #91-014 where study is open.

2.3 To evaluate the predictive value of endorectal coil MRI, the number of positive biopsies on a standard sextant biopsy, PSA, biopsy Gleason sum, and clinical stage on the 2 year actuarial freedom from PSA failure after local field radiation therapy $\pm$ total androgen suppression.

2.4 To determine if the change in the intra- and extra-prostatic tumor extent on the endorectal coil MRI images after 2 months of TAS can be used to assess the subsequent long term freedom from PSA failure to local field radiation therapy and continued TAS (4 months).

3.0 Patient Selection

3.1 <u>Eligibility Criteria</u>

3.101 Histologic Confirmation prostate cancer

3.102 Clinical Stage T1b, T1c, T2a, T2b as per the 1992 AJCC guidelines (Appendix A) Digital rectal exam only and not biopsy or radiographic studies may be used to determine clinical stage.

3.103 No evidence of metastatic disease (-) bone scan and (-) lymph node assessment (CT, MR, or laparoscopic) **OBTAINED WITHIN 6 MONTHS OF ENTRY.** Bone scan is unnecessary in patients with a PSA < 10 ng/ml, and a Gleason Score of $\leq$ 3+4=7.

3.104 Adequate hematologic function: WBC $\geq$ 3,000/ mm^3 (obtained within 1 month of entry)platelet count $\geq 10^5/mm^3$ hematocrit $\geq$ 30%

3.105 An ECOG performance status of 0 or 1. (Appendix B)

3.106 Age $\geq$ 40 years

3.107 A life expectancy of at least 10 years (excluding death related to prostate cancer)

Revised December 14, 1999

3.108 **PSA should be obtained within 3 months of entry, except when a (TURP) Transurethral Resection of the Prostate has been done which has falsely lowered the PSA. In this case, the PSA drawn immediately prior to the TURP will be used as the baseline PSA, even if the PSA exceeds 3 months prior to entry. Patients who previously received Proscar should have PSA repeated 30 days after discontinuation.**

A pretreatment prostate specific antigen (PSA) > 20 ng/ml and ≤ 40 ng/ml

or

a biopsy Gleason score ≥ 7 with a maximum PSA = 40 ng/ml

or

a pretreatment PSA > 10 and ≤ 20 ng/ml and a biopsy Gleason score of 2 - 6 inclusive

or

an endorectal coil MR (+) for either seminal vesicle invasion or extracapsular extension and a pretreatment PSA > 4 and ≤ 10 ng/ml with a biopsy Gleason score of 5 - 6 inclusive

3.109 Signed Informed Consent

3.110 The data manager must be notified prior to patient's randomization.

3.2 Exclusion Criteria

3.21 Prior history of malignancy (except for non-melanoma skin cancer)
3.22 Prior hormonal therapy except for Proscar which must be discontinued at least 30 days prior to registration. Prior chemotherapy for malignant disease.
3.23 Prior pelvic radiotherapy
3.24 Individuals who are unable to tolerate lying still for a 5 - 10 minute radiation treatment because of mental illness or other physical ailment.

4.0 Patient Entry

4.1 Confirm Eligibility and Central Pathology Review (See Section 9.0)
4.2 Baseline studies will be obtained (see Section 6.0)
4.3 Patients will be entered through Marian Loffredo, RN, OCN at (508) - 990 -0588 or by page at (617) - 882 - 9840 (see Section 4.5). Data collection will occur centrally through Heather Hewitt (617) - 632 - 2337 beeper # 1523.
4.4 The Quality Control Center (QCC) [Tel: (617) - 632 - 3761; Fax: (617) - 632 - 2295] will enter and manage all data.
4.5 The following information should be provided to Marian Loffredo, RN, OCN at the time of patient entry:

-Your name and telephone number

-Your name and telephone number
-Protocol name and number
-Date treatment begins
-Patient name
-Date of Birth
-Patient ID number
-Primary Physician and Hospital
-Primary treatment institution
-Confirmation of eligibility (PSA, Gleason sum, clinical stage, CBC, LFT's, ECOG PS, and MRI results if applicable)

5.0 Treatment Plan

5.1 External Beam Radiation Therapy

5.11 Megavoltage linear accelerators ($\geq$ 6 MV) with dose rates between 200 - 400 cGy/min will be used

5.12 In addition to contrast in the bladder and rectum during simulation, diagnostic or treatment planning CT scans can be used to determine the position of the prostate and seminal vesicles

5.13 External beam radiation therapy will commence within one month after simulation and will not start on a Friday.

5.14 25-28 (1.8 Gy) doses of radiation therapy will be delivered to the prostate, seminal vesicles up to a 2 cm margin followed by 8-11 (1.8-2.0 Gy) doses delivered to the prostate and up to a 2 cm margin.

5.15 Radiation therapy will be delivered daily (Monday through Friday, holidays excluded)

5.16 Dose will be calculated to the 95-100% isodose line. This line will encompass the minimal clinical tumor volume (CTV) [1st course CTV = prostate, seminal vesicles and 2 cm margin; 2nd course CTV = prostate and a 2cm margin]

5.2 External Beam Radiation Therapy plus Total Androgen Suppression

5.21 A CT or MRI of the prostate for planning will be taken prior to the start of androgen suppression or the simulation will be done prior to the start of androgen suppression. This will be to guarantee that microscopic and/or macroscopic extracapsular disease is included within our RT planning target volume.

5.22 7.5 mg of Lupron (Leuprolide Acetate) will be given IM at monthly

intervals or Lupron 22.5 mg. IM q 12 weeks or 3.6 mg of Zoladex (Goserlin) will be given SC into the upper abdominal wall every 28 days or Zoladex 10.8 mg q 12 weeks and will begin 2 months prior to the start of radiation therapy (see section 5.1) for a total of 6 monthly injections.

5.23 Flutamide will be administered 250 mg by mouth every 8 hours for 6 months starting at least 24 hours before the first Lupron (Leuprolide Acetate) or Zoladex (Goserelin) injection.

5.24 Two months of androgen ablative therapy with Lupron (Leuprolide Acetate) and Flutamide or Zoladex (Goserelin) and Flutamide will be given prior to external beam radiation therapy. The first course of flutamide will start at least 24 hours before the first Lupron (Leuprolide Acetate) or Zoladex (Goserelin) injection.

5.25 Radiation therapy will commence on Day 60 +/- 1 week and will not start on a Friday

5.26 Two months of androgen ablative therapy with Lupron (Leuprolide Acetate) and Flutamide or Zoladex (Goserelin) and Flutamide will be given during the 2 month course of radiation therapy

5.27 Two months of androgen ablative therapy with Lupron (Leuprolide Acetate) and Flutamide or Zoladex (Goserelin) and Flutamide will be given after the completion of external beam radiation therapy.

5.3 Quality of Life Assessment

5.31 Patients enrolled in the trial will be asked to participate in the quality of life study in patients with prostate cancer that is currently in place throughout the Harvard Medical School affiliated Institutions [DFCI Protocol #91-014]. This study is designed to assess the quality of life and treatment related toxicity in patients undergoing treatment for prostate cancer. This is a patient questionnaire based study. This pertains only to patients enrolled in sites where DFCI Protocol #91-014 is open.

5.4 Adverse Reactions and their management

5.41 Anticipated Toxicity:

5.411 RADIATION THERAPY:

All patients are seen weekly by their radiation oncologist during radiation therapy. Any reactions regarding radiation therapy will be recorded; Appendix D may be used. The related morbidity is discussed with the patient using a separate radiation therapy consent form. *Radiotherapy* may cause reddening or tanning of the skin, hair loss in the treatment

area, temporary fatigue, nausea, diarrhea, abdominal cramps, bladder irritation, and in some cases permanent impotence. There is a small probability (1% - 3%) of injury to the bladder, urethra, bowel and other tissues in the pelvis or abdomen. Common immediate side effects include fatigue, skin redness and irritation in the peri-anal and gluteal folds.

5.412 TOTAL ANDROGEN SUPPRESSION

The related morbidity is discussed with the patient using a separate medical oncology consent form. Common immediate side effects include fatigue, hot flashes, and impotence.

5.42 Toxicity Management

We expect that most side effects associated with radiation therapy and total androgen suppression to be controllable. The use of routine skin care and a low residue diet will be employed routinely and will temporize most of the acute side effects. Liver function tests will be obtained at the time of study entry for baseline purposes and then 2 weeks after the start of flutamide and then monthly until the 6 month drug course is completed.

5.421 Lupron (Leuprolide Acetate)

Mechanism of action of Lupron (Leuprolide acetate)

Lupron (Leuprolide acetate) is a synthetic decapeptide analogue of Luteinizing Hormone Releasing Hormone (LHRH). It acts as a potent inhibitor of pituitary gonadotropin secretion when administered in the biodegradable formulation. Chronic administration of the drug leads to sustained suppression of the pituitary gonadotropins. Consequently serum levels of testosterone fall to castrate levels 2 - 4 weeks after the initiation of therapy. During routine screening of Lupron (Leuprolide Acetate), no significant pharmacological activity was apparent in the cardiovascular system, respiratory, central nervous, renal, metabolic, coagulation, or gastric acid secretory systems. The acute toxicity of Lupron (Leuprolide Acetate) has been found to be very low in relation to its pharmacolgic potency. Studies have shown that serum levels of testosterone can be reduced and maintained within the castrate level resulting in objective evidence of tumor regression. Other than the occasional transient worsening of cancer symptoms (tumor flare in 1% - 5% of patients) due to an initial temporary rise in testosterone levels on initiating therapy, no significant toxicity apart from that attributed to castration (hot flashes, decreased erections, impotence) has been reported. Reports show that the incidence of localized or generalized rash with patients receiving Lupron (Leuprolide Acetate) is 6%. There have been no reports of bronchospasm in the United States Clinical Trials Program. In general, allergic reactions have been extremely uncommon

with Lupron (Leuprolide Acetate) therapy. There have been isolated reports of urethral obstruction, uriticaria, or spinal cord compression. Shortness of breath, cardiac arrhythmia, hyperglycemia, back pain, acute kidney failure, pneumonia, confusion, and weakness were reported in three men. No episodes of anaphylaxis as result of Lupron (Leuprolide Acetate) therapy have occurred in the past.

5.422 Zoladex (Goserelin):

Mechanism of action of Zoladex (Goserelin)

Zoladex (Goserelin) is a synthetic decapeptide analog of gonadotropin releasing hormone. It inhibits gonadotropin production, thus resulting in testicular regression. Worsening of signs and symptoms associated with prostate cancer, such as bone pain in patients with metastatic disease, may occur during the first month of therapy. Patients may experience hot flashes, decreased erection, and impotence. Less frequent toxicities are gynecomastia and pain at the injection site. Cardiovascular or cerebrovascular accidents and skin rashes have rarely been reported.

5.423 Flutamide:

Mechanism of Action of flutamide:

Flutamide exerts its antiandrogenic action by inhibiting androgen uptake and/or inhibiting nuclear binding of androgen in target tissues such as the prostate.

The reported side effects of flutamide include diarrhea, anemia, and mild elevation of SGOT without alteration of the serum bilirubin and without clinical manifestations. Refer to the package insert for additional information. A high percentage of patients treated with flutamide alone developed gynecomastia within 2 - 8 months. One death from liver failure occurred in RTOG 92-02.

5.424 Dose Modification Schedule:

If a patient experiences gastrointestinal discomfort (cramps, diarrhea) prior to the initiation of radiotherapy, flutamide will be withheld until the side effects subside and then reintroduced at a dose of 250 mg/day increasing the dose (at 3 day intervals) to 500 mg/day then 750 mg/day as tolerated. If a patient experiences a
rise in the SGOT or SGPT, then flutamide will be withheld until the liver function tests normalize. Once normalization occurs flutamide can be restarted at 375 mg/day in 3 divided doses. If the liver function tests become elevated again then flutamide will be permanently discontinued. Once elevation of the liver function tests has been documented the liver

function tests should be obtained every two weeks until two consecutive sets of normal LFT's are obtained. Then LFT's should be checked on a monthly basis until the therapy with flutamide is completed.

5.425 Adverse Reaction Reporting:

5.4251 The following serious adverse drug reactions(ADR) attributed to commercial agent(s) used in this study should be reported immediately to Dr. Anthony D'Amico [(508) - 979 - 5858] and Dr. Phillip Kantoff [(617) - 632 - 3466], the radiation and medical oncology study chairs respectively.

5.4252 Any ADR which is both serious (life threatening, fatal) and unexpected

5.4253 Any increased incidence of a known ADR which has been reported in the package insert or the literature

5.4254 Any death on study if clearly related to the commercial agent(s).

5.4255 The ADR report must be documented. The study flow sheet (Appendix D) may be used.

5.43 Criteria for removal from study:

5.431 Patient decision to withdraw from the study
5.432 Patient noncompliance with the requirements of the protocol
5.433 A patient may be removed from the study if it is believed that the constraints of the protocol are detrimental to the patient's health or the ability to deliver the planned radiation therapy.

6.0 Required Data

6.1	Baseline	Every 3 months	At time of PSA failure
History and Physical Exam	X	X	X
Diagnostic prostate biopsy[1]	X		
PSA[2]	X	X	X
Bone Scan[7]	X		X
Lymph node assessment[3]	X		X
Endorectal coil MRI[4]	X		
ECOG performance status	X		X
WBC, hematocrit, platelet count	X		
Liver Function Tests[5]	X		
Quality of Life Assessment[6]	X		
Progression		X	X
Survival		X	X

[1] A Gleason pattern score of prostate tumor histology is requested. The method of diagnostic biopsy should be documented (i.e. transrectal, transurethral, or transperineal).

Central Pathology Review of all original prostate biopsy material is required and will be performed by Dr. Andrew Renshaw at the Brigham and Women's Hospital (See section 9.0)

[2] For patients enrolled into the endorectal coil MRI arm who are randomized to the radiation and TAS arm, a PSA will be obtained on Day 60 prior to the delivery of the first radiation treatment. The 60 day PSA will not be used in determining failure or nadir.

The first post-treatment PSA will be obtained 3 months after the end of radiation treatments in both arms.

Once a patient has failed, PSA and Follow-up should be at the discretion of the physician.

[3] Lymph node assessment can be a CT or MRI of the pelvis or a pelvic lymph node sampling performed open or laparoscopically

[4] Endorectal coil MRI will be obtained in patients with a pretreatment PSA > 4 - 10 ng/ml (Hybritech scale) and biopsy Gleason sum 5 - 6 inclusive and will be optional in all other patients. A second endorectal coil MRI will be obtained after 2 months of androgen deprivation in the group of patients with PSA > 4 - 10 ng/ml and

biopsy Gleason sum 5 - 6. It will optional in all other patients.

[5]Liver function Tests will be obtained at the time of study entry for baseline purposes and then 2 weeks after the start of flutamide and then every month until the flutamide is discontinued. If the flutamide is discontinued because of SGOT or SGPT elevation, liver function tests should be obtained every two weeks. Once the liver function tests normalize and the patient is restarted on 125 mg capsules of flutamide TID. Then the liver function tests should be obtained every two weeks until two consecutive sets of normal LFT's are obtained. Then LFT's should be checked on a monthly basis until flutamide therapy is completed. (See section 5.423)

[6]Patient will be asked to participate in the quality of life study in patients with prostate cancer [DFCI Protocol # 91-014]. Participation in this study requires a separate consent form. Patients are asked to complete the questionnaire prior to treatment, and at follow-up times as outlined in DFCI Protocol # 91-014. This pertains only to patients enrolled in sites where DFCI Protocol #91-014 is open.

[7]Bone Scan is unnecessary in patients with a PSA < 10 ng/ml, and a Gleason Score ≤ 3+4=7.

For baseline studies the following time intervals will be accepted prior to registration:

1 month: WBC, platelets, hematocrit, LFT's

3 months: PSA

6 months: (Lymph node assessment) CT or MR or Lap of the pelvic, bone scan
*Bone Scan is unnecessary in patients with a PSA < 10 ng/ml, and a Gleason Score ≤ 3+4=7.

6.2 Data Collection

**Form**		**SubmissionTime Guidelines**
On-Study Form		≤ 1 month of entry
Treatment Form		
(left side of form)	every week during radiation therapy	≤ 1 month after XRT
(right side of form)	monthly for 6 months	≤ 1 month after TAS
Follow-up Form*	every 3 months for 2 years and every 6 months thereafter	≤ 1 month after follow-up
60-Day MRI Form		≤ 1 month after MRI
Flutamide Complication Form		≤ 1 month after complication

*Once a patient has failed, PSA and Follow-up should be at the discretion of the physician.

6.21 The weekly Treatment Form will be used to assess side effects from radiation therapy and total androgen suppression.

7.0 Modality Review

7.1 PSA failure

The major endpoint of this study is freedom from PSA failure in patients receiving radiation therapy versus those patients receiving radiation therapy plus total androgen suppression. PSA failure is defined as two consecutive rising PSA's obtained after a nadir value has been reached. The time of PSA failure is taken after two rising PSA's have been documented. The time of PSA failure is defined as the time the first rising PSA, (a rise is considered $\geq$ 0.2 ng/ml). Biochemical failure is also defined as any rise in one PSA if one of the following apply: 1) positive Bone Scan, 2) positive CT or MRI of the pelvis, 3) if salvage treatment is begun. Time zero is the time of randomization. If the PSA never decreases at least 0.2 ng/ml, then the time of PSA failure is defined to be zero.

7.2 Local Control

Local Control will be measured as:

Clinical local control as judged by digital rectal examination (Section 7.21).

7.21 Digital Rectal Examination:

Prostate and prostate tumor dimensions in cm may be calculated from the physical exam and recorded on the diagrams found on the data collection forms for initial and follow-up evaluations of the patient. Appendices C and E may be used.

7.211	Complete Response (CR):	Complete disappearance of clinical evidence of disease per DRE.
7.212	Partial Response (PR):	Tumor regression but still evidence of palpable disease.
7.213	Stable Disease (SD):	No change in tumor size
7.214	Progressive Disease(PD):	Clinical evidence in the prostate gland of disease progression.

7.22 Biochemical Control

The PSA level generally decreases after treatment and 90% of patients have reached their nadir by 1 year. Therefore, PSA failure is defined as:

7.221 Two consecutive rising PSA's ≥ 0.2 ng/ml obtained after a nadir value has been reached. PSA failure is also defined as any rise in one PSA if one of the following apply: 1) positive Bone Scan, 2) positive CT of the pelvis, 3) if salvage treatment is begun.

Combination Radiation Therapy and TAS PSA failure will be defined as a PSA greater than 1.0 ng/ml and rising. A rise is considered as two consecutive rises of ≥ 0.2 ng/ml above 1.0 ng/ml, after the PSA plateau following the discontinuation of TAS.

7.23 Distant Control

Upon noting a biochemical failure as per section 7.221, a bone scan shall be obtained and if positive for metastatic disease then the time of distant failure will be defined at the time of a positive bone scan. The time to distant failure will continue to be assessed even after the patient is off study, if a positive bone scan is obtained at a time other than the time of PSA failure, or if it occurs anytime after PSA failure.

7.24 Regional Control

Upon noting a biochemical failure as per section 7.221, a CT of the pelvis shall be obtained and if positive for metastatic nodal disease then the time of regional failure will be defined at the time of the positive CT scan. The time to regional failure will continue to be assessed even after the patient is off study, if a positive CT scan of the pelvis is obtained at a time other than the time of PSA failure, or if it occurs anytime after PSA failure.

7.25 Radiologic Response

In patients having an endorectal coil MRI at baseline and after 2 months of total androgen suppression response criteria is as follows:

Complete Response (CR):	Complete absence of previously noted intra- and extra-prostatic signal abnormalities consistent with tumor
Partial Response (PR):	Abnormal MRI signal that is greater than 50% of the product of the two largest perpendicular dimensions of the original prostate signal abnormality
Stable Disease (SD):	No change in abnormal MRI signal abnormalities that are consistent with tumor

Progressive Disease(PD): Clinical evidence in the prostate gland of disease progression or recurrence as measured by at least a 25% or greater increase in the product of the two perpendicular diameters of the abnormal MRI signal

7.26 Initial Biochemical Response in patients receiving radiation therapy and TAS

The percent decrease in PSA between the baseline measurement and the value obtained pretreatment on day 60 (first day of radiation) will be used to assess the biochemical response to 2 months of TAS, only in patients having the repeated MRI endorectal coil 60 days after baseline or first day of radiation therapy.

7.3 Toxicity

7.31 The acute and late normal tissue toxicity will be scored and recorded weekly. Appendix D may be used.

7.311 Acute Toxicity: Defined as occurring within 7 months of randomization.

7.312 Late Toxicity: Defined as occurring at least 7 months after randomization and will be scored according to the RTOG Late Morbidity Scoring Criteria (Appendix F) and the Common Toxicity Criteria (Appendix G).

8.0 Statistics

8.1 General Study Design

The primary objective of this study is to compare freedom from prostate specific antigen (PSA) failure between patients receiving prostatic radiation therapy with or without total androgen suppression. A total of 270 patients will be stratified by PSA, biopsy Gleason score, and the number of positive biopsies and randomized in equal proportions to the two treatment arms. Assuming an accrual of 100 patients per year and an additional 2 years of follow-up after completion of accrual, the entire study is expected to require 4.7 years.

8.2 Sample Size Determination

Sample size calculations are based on the comparison of freedom from PSA failure between the two treatment arms. Assuming the worse arm has a true median time to failure of 2.7 years (i.e., 60% free from PSA failure at 2 years) and the better arm has a true median to failure of 4.8 years (i.e. 75% free from PSA failure at 2

years), that failure distributions are exponential, that a two-sided log rank test will be used at one interim and one final analysis (see Section 8.3), that the annual accrual is 100 patients per year and that 85% of these evaluable (i.e., get follow-up PSA measures), this design will have 80% power. So 270 patients will be accrued over 2.7 years and followed for an additional 2 years after accrual closes.

8.3 Interim Analyses

Two interim analyses and one final analysis will be conducted. The initial interim analysis, planned for one year following the initiation of accrual, will be used to evaluate safety and feasibility only. If their is evidence at this time of insufficient accrual, the study design will be re-evaluated. Standard interim analysis for the purposes of monitoring quality of life and efficacy endpoints will be performed at approximately 3.0 and 5.0 years following initiation of accrual (the last analysis corresponding to the final analysis of the study). A standard O'Brien-Fleming group sequential boundary will be used at each analysis to determine the statistical significance of any observed differences controlling for multiple interim looks. Appropriate adjustments will be made for looks unequally spaced in terms of information time. Results of all three analyses will be presented to the data monitoring committee. The primary endpoint on which monitoring will be conducted is freedom from PSA failure.

9.0 Central Pathology Review

9.1 Central pathology review of diagnostic biopsies are required for this study. Central reviews of previous prostate studies have reported a 34% discrepancy in histological grading.

9.2 The clinical investigator will mail Dr Andrew Renshaw the hematoxylin and eosin (H & E) stained slides and the pathology report.

Andrew Renshaw, MD
Baptist Hospital of Miami
Department of Pathology
8900 N. Kendall Drive
Miami, Florida 33176

Questions regarding the pathology review can be directed to Dr. Renshaw at (305-596-6525).

9.3 All pretreatment biopsies will be assessed for the presence of tumor and graded according to Gleason (see Appendix H).

10.0 Race/Ethnicity

Historically, it was believed that black race in the United States was a negative prognostic factor for adenocarcinoma of the prostate. It has been

suggested that a higher proportion of whites are diagnosed with early stage disease, thereby skewing survival rates.[8-11] Therefore a large retrospective study evaluating 12,907 men diagnosed with prostate cancer and utilizing the Surveillance, Epidemiology, and End Results Program (SEER) database was performed to address this issue. The study concluded that differences in survival rates were not demonstrated by race for men diagnosed with prostate cancer of any stage after age 70 or for men diagnosed with metastatic disease over the age of 60.[12] Because the vast majority of men enrolled in this study will be over the age of 60 (i.e. not surgical candidates), race will not be an issue that needs to be addressed in the stratification schema. There are no data to date that suggest a difference in outcome by ethnicity in the United States. Finally, no difference in treatment related side effects has been observed due to race or ethnicity in this disease.

References

1. D'Amico, A.V., Whittington, R., Malkowicz, S.B., Schultz, D., Schnall, M., Tomaszewski, J.E., Wein, A.: A Multivariate Analysis of Clinical and Pathological Factors which Predict for Prostate Specific Antigen Failure after radical Prostatectomy for Prostate Cancer, J Urol. 154:131-138, 1995.

2. Partin, A.W., Yoo, J., Carter, B., Pearson, J., Chan, D.W., Epstein, J.I. and Walsh,P.C.: The use of prostate specific antigen, clinical stage, and Gleason score to predict pathological stage in men with localized prostate cancer. J. Urol. 150:110,1993.

3. Presti, J.C., Shinohara, K., Hricak, H., White, S., Cher, M., Carroll, P.R.: Clinical Parameters in the Pre-operative Evaluation for Extracapsular extension at radical prostatectomy. J Urol 153: Abstract 801, 429A, 1995.

4. Labrie, F., Cusan, L., Gomez, J.L., Diamond, P., Suburu, R., Lemay, M., Tetu, B., Fradet, Y., Candas, B. Down-Staging of Early Stage Prostate Cancer Before radical Prostatectomy: The First Randomized Trial of Neoadjuvant Combination Therapy with Flutamide and a Luteinizing Hormone-Releasing Hormone Agonist, Urology.44:29-37, 1994.

5. Pummer, K., Crawford, D.E., Daneshgari, F., Andros, B., Pfister, S., Miller G.J. Hormonal Pretreatment does not affect the final pathologic stage in locally advanced prostate cancer. Urology:44, 38-42, 1994.

6. Sullivan, L., Cleave, M., Goldenberg, L., Bruchovsky, N., Jones E.C. Long Term Neoadjuvant Hormonal Therapy Prior to radical Prostatectomy in Localized Prostate Cancer. J Urol:151, 435A, 831, 1994.

7. Pilepich, M.V., Krall, J.M., Al-Sarraf, M, John, M.J., Scotte Doggett, R.L., Sause, W.T., Lawton, C.A., Abrams, R.A., Rotman, M., Rubin, P., Shipley, W.U., Grignon, D., Caplan, R., Cox, J.D. Androgen Deprivation with Radiation Therapy Compared with Radiation Therapy alone for Locally Advanced Prostatic Carcinoma: A Randomized Comparative Trial of the Radiation Therapy Oncology Group. Urology. 45:616, 1995.

8. Boring, C.C., Squires, T.S., and Heath, C.W., Jr. Cancer Statistics for African Americans. CA 42:7-17, 1992.

9. Mettlin, C., Jones, G.W., Murphy, G.P. Trends in prostate cancer in the United States, 1974 - 1990: Observations from the patient care evaluation studies of the American College of Surgeons Commissions on Cancer. CA 43:83-91, 1993.

10. Natarajan, N., Murphy, G.P., Metlin, C. Prostate cancer in blacks: An update from the American College of Surgeons patterns of care studies. J Surg Oncol 40:232-236, 1989.

11. Ries, L.G., Pollack, E.S., Young, J.L., Jr. Cancer patient survival: Surveillance, Epidemiology, and End Results Program, 1973-1979, J Natl Cancer Inst 70:693-707, 1983.

12. Pienta, K.J., Demers, R., Hoff, M., Kau, T.Y., Montie, J.E., Severson, R.K. Effect of age and race on survival of men with prostate cancer in the Metropolitan Detroit Tricounty Area, 1973 - 1987. Urology 45:93-102, 1995.

APPENDIX A

AJCC/UICC STAGING FOR PROSTATE CARCINOMA

DEFINITIONS:

PRIMARY TUMOR (T)

- TX Primary tumor cannot be assessed
- T0 No evidence of primary tumor
- T1 Clinically inapparent tumor not palpable nor visible by imaging
  - T1a Tumor incidental histologic finding in 5% or less of tissue resected
  - T1b Tumor incidental histologic finding in more than 5% of tissue resected
  - T1c Tumor identified by needle biopsy (e.g. because of elevated PSA)
- T2 Tumor confined within prostate*
  - T2a Tumor involves half of a lobe or less
  - T2b Tumor involves more than half of a lobe, but not both lobes
  - T2c Tumor involves both lobes**
- T3 Tumor extends through the prostatic capsule
  - T3a Unilateral extracapsular extension
  - T3b Bilateral extracapsular extension
  - T3c Tumor invades seminal vesicle(s)
- T4 Tumor is fixed or invades adjacent structures other than seminal vesicles
  - T4a Tumor invades any of: bladder neck, external sphincter, rectum
  - T4b Tumor invades levator muscles and/or is fixed to pelvic wall

*Note: Invasion into the prostatic apex or into (but not beyond) the prostatic capsule is not classified as T3, but T2.

**Note: Tumor found in one or both lobes by needle biopsy, but not palpable or visible by imaging is classified T1c.

REGIONAL LYMPH NODES (N)

The regional lymph nodes are the nodes of the true pelvis, which essentially are the pelvic nodes below the bifurcation of the common iliac arteries. They include the following groups (laterality does not affect the N classification): pelvic, NOS, hypogastric, obturator, iliac (internal, external, NOS), periprostatic, and sacral (lateral, presacral, promontory [Gerota's], or NOS)

SOME DISTANT LYMPH NODES

Distant lymph nodes are outside the confines of the true pelvis. They can be imaged using ultrasound, computed tomography, magnetic resonance imaging, or lymphangiography. Aortic (para-aortic, peri-aortic, lumbar), common iliac, inguinal, superficial inguinal (femoral), supraclavicular, cervical, scalene

- NX Regional lymph nodes cannot be assessed
- N0 No regional lymph node metastasis
- N1 Metastasis in a single lymph node, 2 cm or less in greatest dimension
- N2 Metastasis in a single lymph node, more than 2 cm but not more than 5 cm in greatest dimension, or multiple lymph node metastases, none more than 5 cm in greatest dimension
- N3 Metastasis in a lymph node more than 5 cm in greatest dimension

DISTANT METASTASIS* (M)

- MX Presence of distant metastasis cannot be assessed
- M0 No distant metastasis
- M1 Distant metastasis
  - M1a Non-regional lymph node(s)
  - M1b Bone(s)
  - M1c Other site(s)

*Note: When more than one site of metastasis is present, the most advanced category is used (pM1c is most advanced).

Stage	T	N	M	G
Stage 0	T1a	N0	M0	G1
Stage I	T1a	N0	M0	G2, 3-4
	T1b	N0	M0	Any G
	T1c	N0	M0	Any G
	T1	N0	M0	Any G
Stage II	T2	N0	M0	Any G
Stage III	T3	N0	M0	Any G
Stage IV	T4	N0	M0	Any G
	Any T	N1	M0	Any G
	Any T	N2	M0	Any G
	Any T	N3	M0	Any G
	Any T	Any N	M1	Any G

(Taken from the Fourth Edition Manual for Staging of Cancer. American Joint Committee on Cancer, J.B. Lippincott, 1992)

APPENDIX B

ECOG Performance Status

GRADE

0 Fully active, able to carry on all pre-disease performance without restriction [Karnofsky 90-100].

1 Restricted in physically strenuous activity, but ambulatory and able to carry out work of a light or sedentary nature (e.g., light housework, office work) [Karnofsky 70-80].

2 Ambulatory and capable of all self-care, but unable to carry out any work activities. Up and about ≥ 50% of waking hours [Karnofsky 50-60].

3 Capable of only limited self-care, confined to bed or chair ≥ 50% of waking hours [Karnofsky 30-40].

4. Completely disabled. Cannot carry on any self-care. Totally confined to bed or chair [Karnofsky 10-20].

COMMON TOXICITY CRITERIA

1. Toxicity grade should reflect the most severe degree or most abnormal lab value occurring during the evaluated period.

2. Toxicity grade = 5 if that toxicity caused or contributed to the death of the patient.

3. If patient at baseline has grade 1 or greater, do not code unless patient worsens due to toxicity. If there is worsening, code the level to which the patient increases - DO NOT adjust for baseline.

4. Note that for some Toxicity certain grades are not defined and may not be coded, e.g. no grade 3 or 4 Alopecia.

5. Granulocytes (mature cells) refers to segmented neutrophils (Segs, Polys, PMN, Polymorphonuclear leukocytes) plus bands (Staff cells, Stabs). To calculate Granulocyte count multiply the white count by the % bands + % segmented neutrophils.

6. All coded toxicities must be documented and described on accompanying flowsheets.

November, 1995

COMMON TOXICITY CRITERIA

			Grade		
**TOXICITY**	**0**	**1**	**2**	**3**	**4**
**BLOOD/BONE MARROW**					
WBC	$\geq$4.0	3.0 - 3.9	2.0 - 2.9	1.0 - 1.9	< 1.0
PLT	WNL	75.0 - normal	50.0 - 74.9	25.0 - 49.9	< 25.0
Hgb	WNL	10.0 - normal	8.0 - 10.0	6.5 - 7.9	< 6.5
Granulocytes/ Bands	$\geq$2.0	1.5 - 1.9	1.0 - 1.4	0.5 - 0.9	< 0.5
Lymphocytes	$\geq$2.0	1.5 - 1.9	1.0 - 1.4	0.5 - 0.9	< 0.5
**HEMORRHAGE** (clinical)	none	mild, no transfusion	gross, 1-2 units transfusion per episode	gross, 2-4 units transfusion per episode	massive, >4 units transfusion per episode
**INFECTION**	none	mild	moderate	severe	life-threatening
**GASTROINTESTINAL**					
Nausea	none	able to eat reasonable intake	intake significantly decreased but can eat	no significant intake	--
Vomiting	none	1 episode in 24 hrs	2 - 5 episodes in 24 hrs	6 - 10 episodes in 24 hrs	>10 episodes in 24 hrs, or requiring parenteral support
Diarrhea	none	increase of 2 - 3 stools/day over pre-Rx	increase of 4 - 6 stools/day, or nocturnal stools, or moderate cramping	increase of 7 - 9 stools/day or incontinence, or severe cramping	increase of $\geq$10 stools/day or grossly bloody diarrhea, or need for parenteral support
Stomatitis	none	painless ulcers, erythema, or mild soreness	painful erythema, edema, or ulcers, but can eat	painful erythema, edema, ulcers, and cannot eat	requires parenteral or enteral support

TOXICITY	0	1	2	3	4
**LIVER**					
Bilirubin	WNL	--	<1.5 x N	1.5 – 3.0 x N	>3.0 x N
Transaminase (SGOT, SGPT)	WNL	≤2.5 x N	2.6 – 5.0 x N	5.1 – 20.0 x N	>20.0 x N
Alk Phos or 5' nucleotidase	WNL	≤2.5 x N	2.6 – 5.0 x N	5.1 – 20.0 x N	>20.0 x N
Liver – clinical	no change from baseline	--	--	precoma	hepatic coma
**KIDNEY, BLADDER**					
Creatinine	WNL	<1.5 x N	1.5 - 3.0 x N	3.1 - 6.0 x N	>6.0 x N
Proteinuria	no change	1+ or <0.3 g% or <3 g/l	2 - 3+ or 0.3 -1.0 g% or 3 - 10 g/l	4+ or >1.0 g% or >10 g/l	nephrotic syndrome
Hematuria	neg	micro only	gross, no clots	gross + clots	requires transfusion
**ALOPECIA**	no loss	mild hair loss	pronounced or total hair loss	--	--
**PULMONARY**	none or no change	asymptomatic, with abnormality in PFT's	dyspnea on significant exertion	dyspnea at normal level of activity	dyspnea at rest

TOXICITY	Grade 0	1	2	3	4
**HEART**					
Cardiac Dysrhythmias	none	asymptomatic, transient, requiring no therapy	recurrent or persistent, no therapy required	requires treatment	requires monitoring; or hypotension, or ventricular tachycardia, or fibrillation
Cardiac Function	none	asymptomatic, decline of resting ejection fraction by less than 20% of baseline value	asymptomatic, decline of resting ejection fraction by more than 20% of baseline value	mild CHF, responsive to therapy	severe or refractory CHF
Cardiac- ischemia	none	non-specific T-wave flattening	asymptomatic, ST and T wave changes suggesting ischemia	angina without evidence for infarction	acute myocardial infarction
Cardiac- pericardial	none	asymptomatic effusion, no intervention required	pericarditis (rub, chest pain, ECG changes)	symptomatic effusion; drainage required	tamponade; drainage urgently required

TOXICITY	Grade 0	1	2	3	4
**BLOOD PRESSURE**					
Hypertension	none or no change	asymptomatic, transient increase by greater than 20 mm Hg (D) or to >150/100 if previously WNL. No treatment required.	recurrent or persistent increase by greater than 20 mm Hg (D) or to >150/100 if previously WNL. No treatment required	requires therapy	hypertensive crisis
Hypotension	none or no change	Changes requiring no therapy (including transient orthostatic hypotension)	requires fluid replacement or other therapy but not hospitalization	requires therapy and hospitalization; resolves within 48 hrs of stopping the agent	requires therapy and hospitalization for >48 hrs after the stopping agent

TOXICITY	Grade 0	1	2	3	4
**NEUROLOGIC**					
Neuro- sensory	none or no change	mild paresthesias, loss of deep tendon reflexes	mild or moderate objective, sensory loss; moderate paresthesias	severe objective sensory loss or paresthesias that interfere with function	--
Neuro- motor	none or no change	subjective weakness; no objective findings	mild objective weakness without significant impairment of function	objective weakness with impairment of function	paralysis
Neuro- cortical	none	mild somnolence or agitation	moderate somnolence or agitation	severe: somnolence or agitation or confusion or disorientation, or hallucinations or aphasia, or severe difficulty communicating	coma, seizures, toxic psychosis
Neuro- cerebellar	none	slight incoordination, dysdiadokinesis	intention tremor, dysmetria, slurred speech, nystagmus	locomotor ataxia	cerebellar necrosis
Neuro- mood	Anxiety - none depression - none	mild anxiety mild depression	moderate anxiety moderate depression	severe anxiety severe depression	severe agitation suicidal ideation
Neuro- headache	none	mild	moderate or severe but transient	unrelenting and severe	--
Neuro- constipation	none or no change	mild	moderate	severe	ileus >96 hrs
Neuro- hearing	none or no change	asymptomatic, hearing loss on audiometry only	tinnitus	hearing loss interfering with function but correctable with hearing aid	deafness not correctable
Neuro- vision	none or no change	--	--	symptomatic subtotal loss of vision	Blindness

COMMON TOXICITY CRITERIA

	Grade				
**TOXICITY**	**0**	**1**	**2**	**3**	**4**
**SKIN**	none or no change	scattered macular or papular eruption or erythema that is asymptomatic	scattered macular or papular eruption or erythema with pruritus or other associated symptoms	generalized symptomatic macular, papular, or vesicular eruption	exfoliative dermatitis or ulcerating dermatitis
**ALLERGY**	none	transient rash, drug fever <38C, (100.4F)	urticaria, drug fever >38C (100.4F), mild bronchospasm	serum sickness, bronchospasm, req. parenteral meds	anaphylaxis
**FEVER IN ABSENCE OF INFECTION**	none	37.1 – 38.0C<br>98.7 – 100.4F	38.1 – 40.0C<br>100.5 – 104.0F	>40.0C<br>>104.0F<br>for less than 24 hrs	>40.0C (104.0F) for more than 24 hrs. or fever accompanied by hypotension
**LOCAL**	none	pain	pain and swelling, with inflammation or phlebitis	ulceration	plastic surgery indicated
**WEIGHT GAIN/LOSS**	<5.0%	5.0 - 9.9%	10.0 - 19.9%	≥20.0%	--
**METABOLIC**					
Hyperglycemia	<116	116 – 160	161 – 250	251 – 500	>500 or ketoacidosis
Hypoglycemia	>64	55 – 64	40 – 54	30 – 39	<30
Amylase	WNL	<1.5 x N	1.5 – 2.0 x N	2.1 – 5.0 x N	>5.1 x N
Hypercalcemia	<10.6	10.6 – 11.5	11.6 – 12.5	12.6 – 13.5	≥13.5
Hypocalcemia	>8.4	8.4 – 7.8	7.7 – 7.0	6.9 – 6.1	≤6.0
Hypomagnesemia	>1.4	1.4 – 1.2	1.1 – 0.9	0.8 – 0.6	≤0.5
**COAGULATION**					
Fibrinogen	WNL	0.99 - 0.75 x N	0.74 - 0.50 x N	0.49 - 0.25 x N	≤0.24 x N
Prothrombin Time	WNL	1.01 - 1.25 x N	1.26 - 1.50 x N	1.51 - 2.00 x N	>2.00 x N
Partial thromboplastin time	WNL	1.01 - 1.66 x N	1.67 - 2.33 x N	2.34 - 3.00 x N	>3.00 x N

COMMON TOXICITY CRITERIA

*PERFORMANCE STATUS

CALGB (ZUBROD) Performances Status	PSO Normal	PSI Fatigue without significant decrease in daily activities	PS2 Fatigue with significant impairment of daily activities or bedrest <50% of waking hours	PS3 Bedrest >50% of waking hours	PS4 Bedridden or unable to care for self
KARNOFSKY Performance Status %	100 - 90	80 - 70	60 - 50	40 - 30	20-10

CONVERSIONS / FORMULAS

a) **Body Temperature** F° = C° x 9/5 + 32
C° = F° - 32 x 5/9

c) **Granulocyte Count** WBC 2.2; Segs 61 %;
Bands 4%;
2200 x .65 = 1430

b) **Metric measures** I inch = 2.54 cm
2.2 lbs. = I kg

APPENDIX H

GLEASON CLASSIFICATION[1]

Histologic patterns of adenocarcinoma of the prostate

Pattern	Margins Tumor Areas	Gland Pattern	Gland Size	Gland Distribution	Stromal Invasion
1	Well defined	Single, separate, round	Medium	Closely packed	Minimal, expansile
2	Less definite	Single,separate rounded but more variable	Medium	Spaced up to one gland diameter, average	Mild, in larger stromal planes
3	Poorly defined	Single,separate more irregular	Small medium, or large	Spaced more than one gland diameter, rarely packed	Moderate, in larger or smaller stromal planes
or 3	Poorly defined	Rounded masses of cribriform or papillary epithelium	Medium or large	Rounded masses with smooth sharp edges	Expansile masses
4	Ragged, infiltrating	Fused glandular masses or "hypernephroid"	Small	Fused in ragged masses	Marked,through smaller planes
5	Ragged, infiltrating	Almost absent, few tiny glands or signet ring	Small	Ragged anaplastic masses of epithelium	Severe between stromal fibers or destructive
or 5	Poorly defined	Few small lumina in rounded masses of solid epithelium central necrosis	Small	Rounded masses and cords with smooth sharp edges	Expansile masses

The Gleason Classification is a system of histologic grading based on over-all pattern of tumor growth at relatively low-magnification (40 to 100x). Five patterns of growth are recognized and numbered in order of increasing malignancy. Because of histologic variation in the tumor, two patterns are recorded for each case, a primary or predominal pattern and a secondary or lesser pattern.

The Gleason Score is the sum of the primary and secondary pattern. If only one pattern is present, the primary and secondary pattern receive the same designation.

(Primary = 2, Secondary = 1, Gleason = 3)
(Primary = 2, Secondary = 2, Gleason = 4)

1. Gleason, D.F. et al: Prediction of prognosis for prostatic carcinoma by combined histologic grading and clinical staging. J Urol 111:58, 1974.
