# Supplementary material for: Association of Increased Prostate-Specific Antigen Levels After Treatment and Mortality in Men With Locally Advanced vs Localized Prostate Cancer: A Secondary Analysis of 2 Randomized Clinical Trials
Source: JAMA Netw Open. 2021 May 17;4(5):e2111092. doi: 10.1001/jamanetworkopen.2021.11092 (PMC8129819; doi:10.1001/jamanetworkopen.2021.11092)
Supplement: Supplement 3. — EORTC 22961 Trial Protocol [file jamanetwopen-e2111092-s003.pdf]

EORTC Radiotherapy Group
EORTC Genito-Urinary Tract Cancer Group

Long term adjuvant hormonal treatment with LHRH analogue versus no further treatment in locally advanced prostatic carcinoma treated by external irradiation and a six months combined androgen blockade - A phase III study

EORTC protocol 22961

Study coordinator (Radiotherapy group):

Pr. Michel Bolla **(1)**
Phone: + 33 476765506
Fax: + 33 476765629 // 7555
e-mail: MBolla@chu-grenoble.fr

Study coordinator (Genito-Urinary group):

Dr. Th. M. de Reijke **(2)**
Phone: + 31 20 5666004
Fax: + 31 20 6919647
e-mail: T.M.deReyke@amc.uva.nl

March 1996	**PRC outline approval**	
December 1996	**PRC full protocol approval**	Version 1.0
November 01, 1997	**First amendment**	Version 2.0
July 19, 1999	**Second amendment**	Version 3.0
December 01, 2003	**Third amendment**	Version 4.0

©Copyright EORTC 2003

Contact addresses

**Writing committee:**	Michel Bolla **(1)**, Th. de Reijke **(2)**, Olav Dahl **(3)**, Jean Bernard Dubois **(4)** Abraham Kuten **(5)**, Dionisio Gonzalez **(2),** Jean Claude Horiot **(6)**, René Mirimanoff **(7)**, Guy Storme **(8)**, Urs Studer **(9)**
**Study coordinator (Radiotherapy group):**	Pr. Michel Bolla **(1)**<br>Phone: + 33 476765506<br>Fax: + 33 476765629 // 7555<br>e-mail: MBolla@chu-grenoble.fr□
**Study coordinator (Genito-Urinary group):**	Dr. Th. M. de Reijke **(2)**<br>Phone: + 31 20 5666004<br>Fax: + 31 20 6919647<br>e-mail: T.M.deReyke@amc.uva.nl□
**Data manager:**	Marianne Pierart **(10)**<br>Phone: +32 2 774 16 03<br>Fax: +32 2 771 38 10<br>e-mail: mpi@eortc.be
**Statistician:**	Laurence Collette **(10)**<br>Phone: +32 2 774 16 69<br>Fax: +32 2 771 38 10<br>e-mail: lco@eortc.be
**Quality of Life:**	**Andrew Bottomley (10)**<br>Phone: +32 2 774 16 61<br>Fax: +32 2 779 45 68<br>e-mail: abo@eortc.be
**Health Economics:**	Niels Neymark **(10)**<br>Phone: +32 2 774 16 55<br>Fax: +32 2 772 67 01<br>e-mail: nne@eortc.be
**Quality assurance:**	Geertjan van Tienhoven **(2)**<br>André Dusserre **(1)**, Bernard Davis **(11)**

(1) CHU, Grenoble. **(2)** Academic Medical Center, Amsterdam. **(3)** Haukeland Bergen. **(4)** Centre Val d'Aurelle, Montpellier. **(5)** Rambam Medical Center, Haifa Bat Galim. **(6)** Centre J.F. Leclerc, Dijon. **(7)** CHU Vaudois, Lausanne. **(8)** Oncologisch Centrum, Laarbeeklaan Brussels. **(9)** Inselspital Bern **(10)** EORTC Data Center, Brussels. **(11)** Universitatsspital Zurich.

Table of contents:

1 - BACKROUND AND RATIONALE ... 5
2 - OBJECTIVES OF THE TRIAL ... 6
3 - PATIENT SELECTION CRITERIA ... 6
4 - TRIAL DESIGN AND SCHEMA ... 7
5 - THERAPEUTIC REGIMEN ... 8
6 - REQUIRED CLINICAL EVALUATIONS, LABORATORY TESTS, FOLLOW-UP ... 13
7 - CRITERIA OF EVALUATION, ENDPOINTS ... 14
8 - PATIENT REGISTRATION AND RANDOMIZATION PROCEDURE ... 16
9 - FORMS AND PROCEDURES FOR DATA COLLECTING ... 17
10 - REPORTING ADVERSE EVENTS ... 18
11 - STATISTICAL CONSIDERATIONS ... 19
12 - QUALITY OF LIFE ASSESSMENT ... 19
13 - COST EVALUATION ASSESSMENT ... 21
14 - QUALITY ASSURANCE ... 21
15 - ETHICAL CONSIDERATIONS ... 22
16 - INVESTIGATOR COMMITMENT STATEMENT ... 22
17 - ADMINISTRATIVE RESPONSIBILITIES ... 23
18 - TRIAL SPONSORSHIP/ FINANCING ... 24
19 - TRIAL INSURANCE ... 24
20 - PUBLICATION POLICY ... 25
21 - ADMINISTRATIVE SIGNATURE ... 25
22- LHRH ANALOGUE, DRUG DELIVERY ... 25
23 - LIST OF PARTICIPANTS ... 26
24 - REFERENCES ... 27

Table of appendices:

Appendix 1 : TNM classification of prostate cancer (UICC 1992)

Appendix 2 : WHO Performance status scale

Appendix 3 : Trial Schema

Appendix 4 : Eligibility Checklist

Appendix 5 : Informed consent statement

Appendix 6 : Patient information sheet

Appendix 7 : Flow chart of timing of controls and modality of follow-up

Appendix 8 : Toxicity grading scale

Appendix 9 : Quality of life questionnaire (QLQ-C30)

Appendix 10 : Hospital visit form, Protocol treatment period

Appendix 11 : Declaration of Helsinki

1 Background and rationale

External irradiation alone does not achieve a good local-regional control and progression free survival in locally advanced carcinoma of the prostate (T2c, T3/T4) : cure rates are decreasing with time. Progression free survival based upon biochemical values is even lower (1). Failures are either local or extra pelvic. Three dimensional conformal radiotherapy (which is still under development and evaluation) represents a progress but can only be of benefit to patients with small tumors without pelvic lymph node involvement (2). Results from the RTOG showed that both neoadjuvant hormonotherapy (protocol 86-10) and adjuvant hormonotherapy (protocol 85-31) have significantly improved local control and progression free survival compared to conventional radiotherapy alone. Nevertheless, the optimal hormonal therapy for locally advanced prostate cancer remains controversial.

In protocol 86-10, a neo-adjuvant hormonotherapy with flutamide (250 mg p.o. TID) and Zoladex (3.6 mg monthly), was initiated 2 months prior to start radiotherapy and was stopped at completion of radiotherapy. It resulted in a decreased cumulative incidence of local progression (p=0.001) and in an improvement of progression free survival (p=0.001) for patients with large T2, T3 and T4 prostate tumors with a median follow-up of 4.5 years (3).

In protocol 85-31, adjuvant hormonotherapy with Zoladex was started at the end of the radiotherapy and continued indefinitely, in stage A2/B with regional lymph node involvement (D1), clinical stage C with or without evidence of nodal involvement and pT3 after radical prostatectomy. Local control (p=0.001) and progression free survival (p=0.001) were improved (4).

In RTOG protocol 92-02, the same neoadjuvant combined androgen blockade (CAB) as in protocol 86-10 was given 2 months before radiotherapy and followed during radiotherapy. It was compared to a neoadjuvant TAB 2 months before and during radiotherapy, followed by an LHRH analogue for 2 years in patients classified T2c, T3,T4. This trial was closed in april 1995, and the results are not yet available. The total dose delivered to the prostate was 65-70 Gy for stage T2c, and 67.5-70 Gy for stages T3 and T4.

EORTC 22863 protocol, opened in 1987 and now closed, accrued 415 patients; it addressed the role of a chemical castration by an LHRH analogue (Zoladex) starting at the onset of radiotherapy and followed 3 years in T1/T2 grade 3, and T3/T4 (5).
A steroidal antiandrogen was delivered per os for a month, and at least one week before the first injection of Zoladex, to inhibit the rise of LHRH. The second analysis shows, with a median follow-up of 45 months, a significant increase in local control ($p<0.001$), progression free survival ($p<0.001$), metastases free survival ($p<0.001$), and overall survival (p=0.001). Therefore external irradiation and adjuvant hormonotherapy with LHRH analogue has to be considered as the reference arm in the context of a new trial on locally advanced prostate carcinoma (6).

Can we improve hormonal treatment? Two randomized studies have shown a small, but significant survival benefit in patients with metastatic disease when a combined androgen blockade (CAB) using an LHRH analogue and an antiandrogen over standard management with either orchiectomy or LHRH monotherapy. This advantage was larger in patients with minimal metastatic disease (7); a survey of 22 randomized trials was negative (8). It should be reminded that these trials included metastatic cancer whose hormonodependency might have been decreased with mutagenic instability. After medical castration, the intra prostatic concentration of dihydrotestosterone (DHT) remains at approximately 40% of the measured level in healthy individuals (7); the residual secretion of

testosterone from the adrenal glands is responsible for the stimulation of the prostatic cancer cells, after the transformation of testosterone in DHT by the 5 alpha reductase enzyme. Hence, per os administration of a non steroidal antiandrogen is highly recommended to prevent the interaction of DHT with the prostatic androgen receptor.

Consequently, patients with locally advanced disease staged T1c-T2a-b/N1-2 or pN1-2 and T2c-T4/N0-2 who are at a high risk for metastases, should benefit from a CAB as would those with minimal metastatic disease. Last, Quality of life and Health economics considerations are involved in the comparison of a CAB lasting 6 months initiated at the onset of irradiation and followed for a short period after completion of radiotherapy, versus the same CAB followed an additional 2. 5 years more by LHRH analogue. Provided the same treatment outcome would be achieved, a short term adjuvant CAB would improve quality of life and be less expensive. Moreover the relapse is likely to be salvaged :
a short term CAB being stopped prior tumor progression of androgen independant cells, any subsequent tumor growth which would be due to the proliferation of androgen dependant stem cells would again react positively to androgen withdrawal (9).

2 Objectives of the trial: endpoints

To determine the best hormonal scheme to be associated with pelvic radiotherapy in the curative management of prostatic carcinoma eligible for hormonal treatment with regards to :

- Treatment outcome : overall survival (major end-point), clinical disease free survival (clinical and biochemical), local regional control.
- Quality of life : treatment side-effects and sexual function.
- Health economy : cost effectiveness ratio.

3 Patient selection criteria

3.1 Before the start of radiotherapy (Registration)

To be eligible for randomization, patients must be registered in the protocol immediately prior to starting the radiotherapy and CAB treatments (amendement I to the protocol, November 3,1997). The patients who would have started the treatment before November 3. 1997, remain eligible but should be registered as soon as possible.

3.1.1 Inclusion criteria

Patients must fulfill all of the following criteria to be eligible for admission to the study :

3-1-1-1 Histologically confirmed adenocarcinoma of the prostate.
3-1-1-2 T1c-T2a-b, N1-2 or pN1-2 (after pelvic lymphadenectomy).
3-1-1-3 T2c-T4, N0-2 (UICC 1992) (Appendix 1)
3-1-1-4 WHO performance status 0, 1, 2. (Appendix 2)
3-1-1-5 There is no limit of age.
3-1-1-6 No distant metastases.

3-1-1-7 PSA is mandatory, and must be < 160 ng/ml (Hybritech or < 40xN with N=upper normal limit), before the start of CAB
3-1-1-8 The haemoglobin must be ≥ 100 g/l, the WBC ≥ 2 x 10^9/l and the platelet count ≥ 100 x 10^9/l within the two weeks prior to registration.
3-1-1-9 Patient's informed consent (Appendix 5,6)

3.1.2 Exclusion criteria

3-1-2-1 Stages T1c/T2a-b with a negative pelvic lymph nodes status assessed clinically or surgically.
3-1-2-2 External iliac lymph node metastasis more than 5 cm in greatest dimension (N3).
3-1-2-3 Lymph node involvement to common iliac and/or periaortic lymph nodes (M1a).
3-1-2-4 Previous or concurrent cancers other than basal cell carcinoma cancer.
3-1-2-5 Any type of treatment for prostate cancer except hormonotherapy given within 3 weeks prior to registration

3.2 Before randomization (Appendix 4)

Additional eligibility criteria have to be fulfilled before randomization.
3-2-1 Patients having received a 6 months combined androgen blockade
3-2-2 Patients not in progression after the 6 months of combined androgen blockade
3-2-3 WHO performance status 0, 1, 2. (Appendix 2)
3-2-4 PSA < 160 ng/ml (Hybritech or < 40xN)

4 Trial design and schema

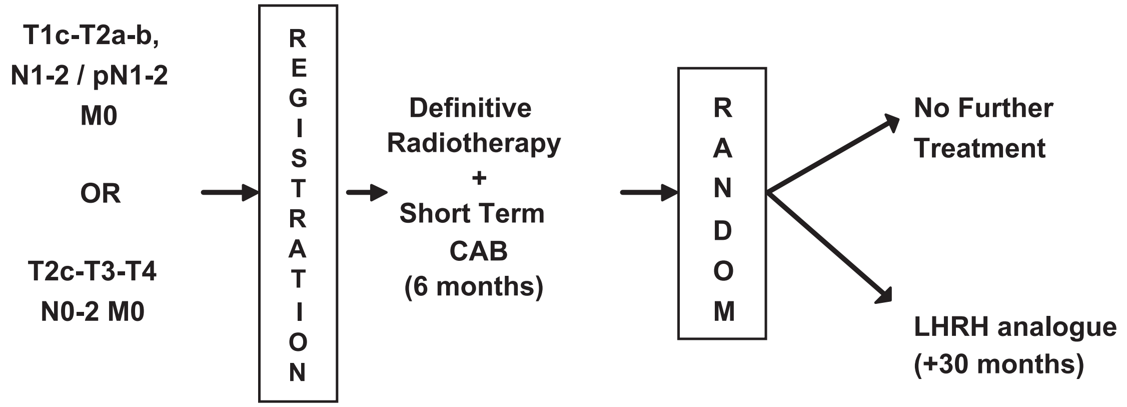

5 Therapeutic regimen

5.1 Protocol Treatment

All patients will receive external irradiation for 5 weeks, followed by a pelvic boost given for two weeks and a six months combined androgen blockade initiated at the onset of external irradiation. Then, all patients will be randomly allocated between :

- no further treatment
- or a 2 years and a half year hormonal treatment with an LHRH analogue given every 4 weeks or 3 months according to the drug conditioning.

5.1.1 Radiation treatment

5.1.1.1 Position of the patient

The patients are usually treated in the supine position; the prone position may only be used when a customized immobilization device is available.
The same position will be reproduced during simulation, CT or contour acquisition and treatment. The reproducibility of the position will be checked by orthogonal laser beams.
Nota Bene : The patient is asked not to void his bladder before each step of treatment planning and before each treatment session, during about 1.5 hours, to keep a filled bladder in order to irradiate less bladder wall and gut.

5.1.1.2 Patient data acquisition

The CT investigations should be made in the treatment position, with a flat table top and with the immobilization device, if applicable. The magnitude of prostate and seminal vesicle mobility is reduced by asking the patient not to empty his bladder before the simulation CT.
In case of 3 D conformal radiotherapy, CT images should be obtained with a 4-5 mm slice thickness, from approximately 3 cm above the superior tips of the seminal vesicles to 3 cm below the prostate. Additional CT images with a slice thickness of 1 cm may be obtained above and below these levels extending from the lower abdomen to the thighs.

5.1.1.3 Volumes of interest

The different volumes of interest should be defined according to the recommendations of ICRU Report 50.

5.1.1.3.1 Clinical target volume I

5.1.1.3.1.1 Extended pelvic fields

Prostate, periprostatic tissues and all known areas of tumor extension, seminal vesicles, external and internal iliac lymph nodes, low part of common iliac lymph nodes.

5.1.1.3.1.2 Small pelvic fields (Amendment)

Centers which usually irradiate with small pelvic fields are allowed to treat in such a way, provided patients are N0, i.e. with no evidence of tumor spread to the pelvic lymphatics. The CTV I will include prostate, seminal vesicles, external and internal iliac lymph nodes.

5.1.1.3.2 Clinical target volume II

It includes prostate, seminal vesicles, periprostatic tissues and all known areas of tumor extension.

5.1.1.3.3 Organs at risk

The bladder and the rectum are the organs at risk. The rectal volume should not be included to more than half of the rectal lumen.
Three dimensionnal treatment planning with patient immobilized and the use of beams eye view technique for conformal blocking are recommended.

5.1.1.4 Simulation procedure

A simulation procedure is mandatory for all fields. Retrograde cystography (with nearly 30cc of fluid) and opacifition of the rectum by thin barium (50cc) are required to outline anatomical structures covered the fields. The position of all shielding blocks should be indicated on the simulation films.
Nota Bene : This may be omitted if the treatment planning system used can calculate DDR/portal images in which organs are clearly visible.

5.1.1.5 Treatment technique

5.1.1.5.1 Treatment parameters

The treatment technique should be isocentric. A four field technique with two AP/PA and two opposed lateral fields is recommended provided the definition of the PTV and the dose homogeneity requirements across the PTV comply with protocol recommendations.
A three field arrangement with one anterior and two lateral fields may be acceptable. A six-field coplanar prostate treatment plan, consisting of one pair of lateral and two pairs of oblique fields is recommended for the boost.

PTV I :
Extended fields : Clinical target volume includes lower part of primitive iliac lymph nodes, external iliac lymph nodes, seminal vesicles and prostate. A security margin of 2 cm is recommanded in all directions.
Small fields (Amendment) : Clinical target volume includes external and internal iliac lymph nodes, seminal vesicles and prostate. A security margin of 2 cm is recommanded in all directions.

PTV II :
Clinical target volume includes prostate and seminal vesicles. A security margin of 1.5 cm with respect to prostate and seminal vesicles must account for prostate movements and inacuracies in beam and patient set-up.

5.1.1.5.2 Required field shaping

PTV I : Cerrobend (Lipovitz's metal) customized blocks are required to spare as much as possible femoral heads, anal canal and rectum.

PTV II : Customized blocks are not advised, except for shielding anal canal on laterals fields if necessary.
In both cases, conventional Cerrobend (Lipovitz's metal) blocking may be replaced by computer-driven multileaf collimators (MLC) for automated beam shaping.
Standard blocks (straight edged) are not allowed.

5.1.1.6 Dose computation

The dose distribution should be shown at the plane through the beam axes, both for PTV I and PTV II. The planning target area in these planes should be outlined. The dose distribution calculation may also be requested at planes 2-3 cm from the cranial and caudal field edges, depending on the shape of the PTV.

5.1.1.7 Equipment

Megavoltage equipment with a nominal photon energy of 5 MV or greater and a minimum source to axis distance of 100 cm is required.

5.1.1.8 Dose prescription, recording and reporting

The dose is specified at the intersection of the beam axes which is referred to as the ICRU reference point. The reported dose values are the doses at the prescription point and at other points specified in the protocol.

5.1.1.9 Dose homogeneity

The absorbed dose should be within a given percentage value of the prescribed dose, i.e. -5% to +5%, according to ICRU 50.

5.1.1.10 Fractionation Schedule

Dose per fraction : 2 Gy.
Number of fraction per day : one.
Number of fractions per week : five.
Total number of fractions : 35.
50 Gy in 25 fractions over 5 weeks for PTV I.
20 GY in 10 fractions over 2 weeks for PTV II.
70 Gy total dose in 35 fractions, overall treatment time equal to 7 weeks.

5.1.1.11 Treatment verification

Portal imaging films must be obtained for PTV I and II during the course of treatment, and should be compared with the simulators films.

5.1.2 Hormonal Treatment

5.1.2.1 Short term hormonotherapy : Combined Androgen Blockade

5.1.2.1.1 Antiandrogens

A non steroidal antiandrogen Flutamide (Eulexine) will be given orally at a dose of 250 mg three times daily for a total daily dose of 750 mg, and will be continued for 6 months.
Another non steroidal antiandrogen Bicalutamide (Casodex) may be used at a daily dose of 50mg, provided this formulation is licensed. In a randomized, double-blind trial involving 813 previously untreated metastatic prostate cancer patients, Bicalutalide was shown to be as effective as Flutamide (daily dose of 750 mg), with a better gastro-intestinal tolerance (10).
To protect from flare, antiandrogen treatment should start one week before the first injection of LHRH analogue.

5.1.2.1.2 Luteinizing Hormone Releasing Hormone Analogue (LHRH-A)

LHRH-A will be used as a 3.75 mg intramuscular monthly depot of Decapeptyl® (Triptoréline), rather than a 3 monthly depot preparation. The first injection of LHRH-A will be given the first day of irradiation, and will be repeated each month for a total duration of six months.

5.1.2.2 Long term hormonal treatment

Patients randomized in arm 2 will receive the LHRH-A for an additional 30 months, with the same chronology of administration, without additional Flutamide (or Bicalutamide); the administration will be stopped in case of disease progression.

From March 1998 Ipsen Biotech company will provide us with the drug Decapeptyl® free of charge. As a consequence this drug has to be used exclusively unless the patient is submitted to an anti-coagulant treatment; in this case a subcutaneous injection of Zoladex® (Goserilin acetate) is advised.

Patients would have started the long term hormonal treatment before the amendment should use Decapeptyl®.

5.2 Expected side-effects and dose modifications

5.2.1 Flutamide

Flutamide is a potent inhibitor of androgen uptake in endocrine-target tissues. Its major metabolite is the alpha hydroxylated derivative and both compounds have a plasma half-life of 5 to 6 hours; 28 % of the drug is excreted in the urine within 24 hours.

Side effects include diarrhea and mild elevation of SGOT without alteration in serum bilirubin and without clinical manifestations. Gynecomastia, even galactorrhea, can develop but usually disappears with discontinuation of treatment.

If during irradiation, the severity of diarrhea exceeds the level commonly observed during pelvic irradiation (i.e. diarrhea requiring parental fluid support), the toxicity will be reported to Flutamide and the drug will be permanently discontinued.

If gastrointestinal disturbances occur after the administration of radiotherapy, and if the level observed exceeds the one observed during irradiation (i.e.diarrhea requiring parenteral fluid support), the toxicity will be related to Flutamide and the drug will be permanently discontinued. If the level of gastrointestinal disturbances is lower, flutamide will be withheld untill the side effects disappear and then reintroduced at a dose of 250 mg/day, increasing the dose to 500 mg/day, then 750 mg as tolerated.

5.2.2 Bicalutamide

Side effects are the same as for Flutamide but, with less diarrhea and less elevated levels of transaminases.
Consequently, Bicalutamide will be withheld in case of gastro intestinal toxicity higher than the one observed with radiotherapy alone and then reintroduced as soon as the symptoms have disappeared.

5.2.3 LHRH Analogues

No significant toxicity has been reported apart from that attributed to castration, hot flushes, decreased erections, and impotence.

5.2.4 Radiation toxicity

Diarrhea.
Rectal urgency , bleeding.
Nausea.
Urinary frequency, dysuria, hematuria.
Acute toxicity will be assessed according to the CTC-NCIC toxicity scale (appendix 8)
Late toxicity will be assessed with the modified EORTC/RTOG scale (appendix 8)

5.3 Guidelines for second line treatment at relapse

5.3.1 Biochemical progression

An increase in PSA by itself does not justify a change of treatment (until documented data of the relapse either local and/or distant are obtained) and is no reason for going off study.
Due to the fact that treating a documented clinical relapse as soon as possible may improve survival, many physicians are keen to treating biochemical relapse, all the more as patients want to be treated because they are afraid by observing a rising PSA without other clinical proof of relapse.
To improve the reliability assurance of the follow-up and to facilitate the evaluation of the results, it is proposed that whenever biochemical relapse prompts for a start of treatment, <u>this second line treatment should only be started whenever the PSA value increases to a value above or equal to 4 ng / ml (if hybritech assay or equivalent, or above the upper limit of normal for other assays).</u> This attitude is not harmful to the patients and gives us the opportunity to better estimate a biochemical relapse.

Every institution should however decide and follow an identical practice for the patients randomized to both treatment groups of the trial. Institutions who wish to treat upon biochemical relapse should do so for all the patients they treat, irrespective of the randomized treatment. Institutions who prefer to wait for a local or distant progression should do so for all their patients, irrespective of the randomized treatment.
The guidelines for the type of treatment to be started as as specified in 5-3-2.

5.3.2 Local and/or distant progression

Arm 1 :
Same treatment as in the immediate treatment arm : LHRH-A. In case of no effect or in case of relapse, a non-steroidal anti-androgen has to be added.

Arm 2 :
Complete androgen blockade with a non-steroidal anti-androgen and an LHRH-A or a bilateral orchiectomy .

6 Required clinical evaluations, laboratory tests, follow-up

6.1 Evaluation before the start of radiotherapy

6.1.1 Clinical examination

Tumor size in cm in two dimensions.
WHO performance status.
Sexual activity will be evaluated both before the onset of radiotherapy and complete androgen blockade, and at the time of randomization : ability to have erection, ability to have intercourse.
Gleason grade, WHO histological grading.
General examination.

6.1.2 Imaging studies

Bone scan.
CT of abdomen and pelvis.
Chest X-ray.

6.1.3 Laboratory studies

Blood count.
Serum testosterone level.
Prostatic specific antigen (PSA).
Transaminases level (SGOT, SGPT).
Total bilirubine.
Serum creatinine.

6.2 Evaluation before randomization

Endorectal examination.
Serum PSA determination.
The feasability and the results of protocol 22863 suport the choice of two criteria serum PSA for the assessment of baseline and follow-up status : after a 6 months hormonal treatment, PSA level is always under the upper limit of normal and the prostatic lodge is flattened.

6.3 Follow-up (Appendix 7)

6.3.1 Clinical follow-up

Every 6 months during the five first years.
Annually the remainder of the patient's life.

6.3.2 Laboratories studies

PSA at each follow-up.
Serum testosterone in arm 2.

6.3.3 Imaging studies

Chest X-ray, Technetium bone scan, CT scan of pelvis and abdomen are not mandatory every year, but are required, should there be a clinical and/or biochimical (PSA) suspicion of progression. CT scan or MRI should be performed when the interpretation of bone scan is difficult.

7 Criteria of evaluation, endpoints

7.1 Criteria of evaluation

7.1.1 Survival

Survival is defined as the time from the date of randomization to the date of death from any cause or to the date of last follow-up; effort should be made to document the cause of death.

7.1.2 Disease free survival

Disease free survival is defined from the date of randomization to the earliest occurrence of either death, local and /or regional failure, distant metastases or the date of the last follow-up.

7.1.3 Time to first treatment failure

Time to first treatment failure is measured from the date of randomization to the date of clinical progression or the date of PSA progression or the date of most recent follow-up.

7.1.4 Time to local progression

Time to local progression is measured from the date of first treatment to the date of documented local progression as assessed by the following symptoms :

- Palpable enlargement of an existing abnormality or regrowth of a previously regressed prostate gland must be considered as a disease progression or recurrence when there is a 25% or greater increase in the product of the two largest diameters of the prostate, and must be documented by a positive biopsy to be considered as failure or relapse.

- The development of an obstructed ureter constitutes evidence of progression.

- Urethral obstruction or bleeding necessitating a trans-urethral resection constitutes evidence of progression only if the resected tissues demonstrate viable malignancy.

7.1.5 Imaging and/or Clinical Criteria of Metastases

Bone metastases are detected by the appearance of new hot spots on bone scan.

Pulmonary metastases are detected by chest X-ray and if necessary by C.T. scan.

Liver metastases are specified by ultrasound or C.T. scan.

Soft tissue metastases are identified either clinically or by means of various radiological procedures (ultrasound or C.T. scan) or from laparotomy performed for any reason during the follow-up. Metastases should be proven by biopsy if accessible.

Pelvic or para-aortic lymph node progression is demonstrated by ultrasound or C.T. scan and should be proven by biopsy (fine needle biopsy or surgical biopsy); nevertheless, sonographic or imaging evidence of inaccessible lymphadenopathy > 2 cm in maximum diameter is in itself sufficient to constitute progression.

7.1.6 Biochemical Criteria of Progression

A biochemical progression is every increase of serum PSA above the upper limit of the nadir, which is 1.5 ng/ml (for hybritech assays or equivalent*), after a period of normalisation, confirmed by a further confirmatory serum PSA not less than 3 months apart. **The ASTRO criteria of PSA relapse (Int J Radiation Oncology Biol Phys 1997;37:1035-1041) are not applicable for patients who have received hormonal treatment 6 months or either 36 months; it is the reason why we have recommended to consider a PSA value higher than 1.5ng/ml (for hybritech assays, assuming an upper limit of normal of 4 ng/ml*) as a criteria of biochemical failure, as we did for trial 22863. (Bolla M, et al.N Engl J Med 1997;337:295-300.)**
***The equivalent to this limit of 1.5 ng/ml for other non-hybritech assays is 3xUNL/8.**

7.2 Endpoints

7.2.1 Primary endpoint

overall survival.

7.2.2 Secondary endpoints

Clinical disease free survival,
Clinical disease free interval,
Local regional control,
Acute toxicity according to the CTC-NCIC Scale (Appendix 8)
Late toxicity according to the modified EORTC/RTOG scale, (Appendix 8)
Quality of life and sexual function, according to EORTC QLQ-C30 (Appendix 9)
Cost effectiveness study (Appendix 10).

8 Patient registration and randomization procedure

8.1 Registration

Patients must be registered to the trial at the time of starting the Radiotherapy and the first 6 months of CAB treatment. The informed consent should be obtained before registering a patient in this trial. At the time of registration, the following information will be asked:

1. WHO performance status?
2. Histologically proven carcinoma of the prostate?
3. Clinical T category ?
4. Gleason ?
5. Clinical N-category?
6. Pathological N category, (after lymphadenectomy), if appropriate ?
7. Lymph nodes involvement to common iliac and/or periaortic lymph nodes?
8. Presence of distant mets ?
9. Other previous or concurrent maligancies?
10. Any prior treatment for prostate cancer except hormonotherapy given within 3 weeks prior to registration?
11. WBC($x10^9$/l)
12. Platelets (10^9/l)
13. Hemoglobin (mmol/l or g/l)
14. PSA level (ng/ml)
15. PSA laboratory upper normal limit (ng/ml)
16. Informed consent obtained?
17. Group affiliation of the member registering the patient: RTX or GU

The patient will be assigned a sequence number (seqid)

8.2 Randomization

Six months after the start of CAB treatment (registration), the patient should be randomized between stopping hormonotherapy or continuing LHRH treatment for another 2.5 years. Patients who show progression of the disease are not allowed to be randomized.

The randomization will be centralized in the EORTC Data Center either by internet or EuroCODE, 24 hours a day, 7 days a week or by telephone (32 2 774 16 00) from 9 am to 6 pm Monday to Friday. The randomization will be done using the minimization technique.

The randomization will be stratified by :

- ♦ institution
- ♦ clinical tumoral stage (T1c T2a-b versus T2c-T3-T4)
- ♦ nodal stage (N0 vs (p)N1 vs (p)N2)
- ♦ initial PSA (5xN (20 ng/ml, Hybritech), 5 - 10 xN, > 10 xN (40 ng/ml Hybritech))
- ♦ Gleason (2-7 vs 8-10)

The following information will be asked at the time of randomization of the patient:

1. WHO performance status?
2. Has the patient received 6 months of CAB?
3. Patient in local progression after 6 months CAB?
4. Patient in regional progression after 6 months of CAB?
5. Common iliac or periaortic lymph nodes involvement?
6. Presence of distant mets?
7. Current PSA level (ng/ml)
8. Patient able to understand and fill out QoL form?
9. Baseline QoL form filled out?

The patient will then be automatically and randomly assigned to one of the two treatment arms:
-Arm 1: no further hormonal treatment
-Arm 2: Additional 30 months of LHRH treatment from the date of randomization

9 Forms and procedures for data collecting

All forms are supplied by the Data Center.

9.1 Registration checklist

To be sent at the time of registration

9.2 On study form

To be sent at the time of registration

9.3 Short term hormonotherapy form

To be sent at the time of stopping short term CAB treatment

9.4 Radiotherapy form

To be sent at the end of the radiotherapy treatment

9.5 Randomization checklist

To be sent within one week of randomization

9.6 Follow-up forms

For the randomized patients only, to be sent every 6 months for 5 years after randomization and yearly thereafter and in any case of progression or death.

All patients must be followed until death with follow-up forms despite a first progression of the disease or a change of treatment. All events of progression should be reported.

9.7 Quality of life evaluation

Baseline assessment will be performed at registration (prior to starting treatment, (amendment)) and at randomization (prior to being informed of the randomized treatment). Follow-up assessment will be performed at 6, 12, 24 and 36 months after randomization and a single question (EORTC Health Thermometer) will be asked yearly thereafter (Appendix 9)

9.8 Health Economics Forms

Data will be collected for each treatment and follow-up episode.

10 Reporting adverse events

The acute toxicity will be reported on the radiotherapy form, and the late toxicities will be reported on the follow-up forms. In case of serious adverse events, the Data Center should be informed within 48 hours of occurence and the Data Manager should transmit the information to the study co-ordinator.

10.1 Flutamide

Diarrhea
Mild elevation of SGOT without alteration in serum bilirubin and without clinical manifestations
Gynecomastia, even galactorrhea.

10.2 Bicalutamide

Side effects are the same as for Flutamide but, with less diarrhea and less elevated levels of transaminases.

10.3 LHRH Analogues

Hot flushes, decreased erections, impotence.

10.4 Radiation toxicity

Diarrhea
Rectal urgency , bleeding
Nausea
Urinary frequency, dysuria, hematuria.

11 Statistical considerations

11.1 Number of patients required

External radiotherapy associated to 3 years of adjuvant hormonotherapy is now our reference treatment based upon the results of trial 22863. The aim of the present trial is to evalute if a shorter hormonal therapy gives an equivalent survival. The 5-year survival rate in the reference arm is estimated to be 80 %. Equivalence will be defined as a relative risk not greater than 1.35, which correspond to a decrease of the 5-year survival rate from 80 % to 74 %.

Based upon that hypothesis, a total of 275 deaths will need to be observed to prove the equivalence with a power of 80 % (b=0.2), and a one-sided type I error rate of 5 % (a=0.05). To observe those events, a projected total number of 966 patients should be randomized (483 in each arm) over a period of 5 years; this implies an accrual rate of 193 patients per year. The final analysis of survival will be performed as soon as a total of 275 deaths will be recorded. On the basis of the above hypothesis, this will require a follow-up period of 5 years after the last inclusion.

11.2 Statistical analysis

Survival and progression free survival rates will be estimated according to the Kaplan-Meier technique (11), with a 95% confidence interval. The comparison of the curves will be done using a two-sided logrank test (12). Analysis will be done with an intent to treat policy.

12 Quality of life assessment

12.1 Rationale

From previous studies it is known that hormonal treatment for prostate cancer can produce treatment related side-effects, that have a negative influence on the quality of life of these patients. In this study, quality of life is a secondary endpoint and will be evaluated in a longitudinal design.

The aim of quality of life evaluation in this study is to investigate whether CAB initiated at the start of radiotherapy and followed for six months will have a less negative effect on quality of life as compared to the combination of CAB for six months followed by LHRH-A for 2.5 years.

Health related quality of life is a multidimensional construct, which can be defined as a state of general well being reflecting physical, psychological, and social well being and the control of the disease and/or treatment related symptoms. The aspects of quality of life that are most likely to be affected in this study are treatment related side effects, sexual functioning and overall quality of life.

Since treatment related side effects and sexual dysfunction will gradually disappear once the hormonal treatment is being stopped, it is expected that patients who receive the short term CAB will recuperate earlier from the side-effects, which in turn will have a positive effect on the overall quality of life. If this proves to be the case, then the possible gains in terms of lenght of life will have to be balanced against a prolonged burden of treatment.

12.2 Design of the quality of life evaluation

Quality of life will be evaluated in a long longitudinal design in all patients entered in the study. Assessments will consist of a self administered questionnaire. In this study the EORTC core questionnaire QLQ C 30 version 2.0 will be used (Appendix 9), which is a well-validated and accepted instrument to measure various domains that constitute quality of life. It consists of 30 questions which form 5 functional scales, 3 symptom scales, and a global health/quality of life scale. The remaining single items assess additional symptoms commonly reported by cancer patients.

In addition to the core questionnaire a few questions will be added that focus on treatment related side -effects and sexual functioning. These questions will have the same format as in the core questionnaire.

Since a cost effectiveness analysis will also be performed in this study it is relevant to combine the three outcome measures, i.e. length of life, quality of life and economic aspects of the two treatments arms in one overall outcome measure such as quality and cost adjusted survival by using e.g. a Quality Adjusted Life Years (QALY) model. In order to be able to do this, a valuation of the health related quality of life is needed. For this purpose the EORTC Health Thermometer will be added (a single question).

Baseline assessment will be performed at registration (amendment) and at randomization. Follow-up assessment will be performed at 6, 12, 24 and 36 months after randomization (Follow up visite schedule appendix 7) and will coincide with the schedule follow-up visits of the patient at the hospital. After three years and as long as patients are on-study, they will be asked to fill out the single question of the EORTC Health Thermometer at each follow-up visit.

12.3 Analysis of the quality of life data

In this study the treatment related side-effects and sexual functioning are considered to be the most important domains of quality of life. We anticipate a continuation of the side-effects and problems in sexual functioning in the treatment arm that will receive prolonged LHRH-A. Data from the treatment specific questions and sexual functioning will be compared to assess the percentage of patients in each treatment group which report moderate or severe problems at each point of assessment as compared to baseline assessment.

Descriptive analysis will be performed on the data from the EORTC QLQ-C30 to investigate the stability of the functional scales over time in the two arms. Mean groups scores on the EORTC Health Thermometer will be performed to a utility Time Trade-off score using a power function (13). These adjusted scores will be used as the quality adjusted factor in the cost-utility analysis.

13 Cost evaluation assessment

Knowledge about the expected costs of each of the compared treatment and about the incremental cost-effectiveness of using one of them rather than the other, is an important element in the decision making process anywhere. This decision process is relevant to the adoption of new treatments as well as for the determination of an appropriate rate of reimbursement. Also, an estimate of the overall cost of the treatment of high metastatic risk prostate cancer patients, may be helpful in achieving greater treatment efficiency.

To enable reliable calculation of expected costs and cost-effectiveness of the treatments compared in this trial, information on the day cost drivers will be collected prospectively in all participating centres, simultaneously with the clinical data.

The following data will be collected for each treatment and follow-up episode :

- ♦ dates of hospital admission and discharge, outpatient visits and "one day " hospitalisation
- ♦ the ward and type of bed in which hospitalisation takes place
- ♦ main reason for hospital stay or visit (e.g. treatment, toxicity, late side-effects...)
- ♦ patient travel data

These data will be complemented with additional data obtained from selected centers concerning resource utilisation not covered by the prospective phase as well as unit cost data.

14 Quality assurance

14.1 Control of data consistency

Data forms will be entered in the database of the EORTC Data Center by a double data entry procedure.
Computerised and manual consistency checks will be performed on newly entered forms ; queries will be issued in case of inconsistencies.
Consistent forms will be validated by the Data Manager to be entered in the master database.
Inconsistent forms will be kept "on-hold" until resolution of the inconsistencies.

14.2 On-site quality control : dummy run and in vivo dosimetry

A quality control procedure will be performed after the inclusion of the first five patients by each participating centre. It will consist of a dummy run procedure and dosimetry check aimed at comparing and evaluating the irradiation technique and suggesting corrections when needed to ensure consistency between participating centres.

14.3 Individual case review

After entering a minimum of 20 patients, every center is subject to an individual case review procedure, with the aim of identifying the compliance with eligibility criteria and protocol treatment guidelines as well as thetimeliness and quality of submission of the necessary forms.

14.4 Quality Assurance Committee

A quality assurance committee consisting of the trial coordinators and at least two radiation physicists will organise the quality control and dummy run procedures.

15 Ethical considerations

15.1 Patient protection

The responsible investigator will ensure that this study is conducted in agreement with either the Declaration of Helsinki (Tokyo, Venice and Hong Kong amendments) or the laws and regulations of the country, whichever provides the greatest protection of the patient.
The protocol has been written and the study will be conducted according to the guidelines for Good Clinical Practice issued by the European Union.
The protocol will be approved by the EORTC Protocol Review Committee and by the Local, Regional or National Review Boards.

15.2 Subject identification

A sequential identification number will be automatically attributed to each patient registered in the trial. This number will identify the patient. However, to avoid identification errors, patients' initials (maximum of 4 letters), date of birth and local chart number will be reported on the case report forms.

15.3 Informed consent

All patients will be informed of the aims of the study, the possible adverse experiences, the procedures and possible hazards to which he will be exposed. It will be emphasised that the participation is voluntary and that the patient is allowed to refuse further participation in the protocol whenever he wants. This will not have any consequences for the patient subsequent care. Documented informed consent must be obtained for all patients included in the study, before registration at the EORTC Data Centre. This must be done according to the national and local regulatory requirements and the local rules followed in the institution.

16 Investigator commitment statement

Investigators will only be authorised to register patients in this trial when they have returned to the Data Center :

- A commitment statement, indicating that they will fully comply with the protocol, and including an estimation of their accrual.
- Their updated Curriculum Vitae.
- A copy of the letter of acceptance of the protocol by their local ethical committee.

17 Administrative responsibilities

The study cordinator (in cooperation with the Data Center) will be responsible for writing the protocol, reviewing all case report forms and documenting his review on evaluation forms, discussing the contents of the reports with the Data Manager and/or the Statistician, and preparing the publication of the results of the study. He will coordinate the interactions between the groups involved in more specific aspects of the trial (Quality assurance, Health Economics and Quality of life). He will also be generally responsible for answering all clinical questions concerning eligibility, treatment, and evaluation of the patients.

Study coordinators :

Pr Michel Bolla
Radiotherapy Department
CHR DE GRENOBLE - LA TRONCHE
BP 217X
F- 38043 GRENOBLE cedex
FRANCE
Tel : +33 4 76 76 55 06
Fax : +33 4 76 76 56 29
email : Michel.Bolla@chu-grenoble.fr

Dr Theo De Reijke
Department of Urology
Academisch Medisch Centrum
Meibergdreef 9
NL-1105 AZ AMSTERDAM
THE NETHERLANDS
Tel : 00 31 20 566 60 04
Fax : 31 20 691 96 47
Email : T.M.deReyke@amc.uva.nl

EORTC Data Center

The EORTC Data Center will be responsible for reviewing the protocol, collecting case report forms, controlling the quality of the reported data, and generating reports and analyses in cooperation with the Study Coordinator. All methodological questions should be addressed to the EORTC Data Center at the following address :

EORTC Data Center
Avenue Emmanuel Mounier 83, bte 11
1200 BRUSSELS
BELGIUM
Fax: +322 772 35 45

Registration of patients:

Tel.:+32 2 774 16 00
or use EuroCODE direct line:+32 2 772 04 26, PTT network (206) 22 15124
or Internet (telnet to ecvax@eortc.be)

Statistician:

Laurence Collette
Tel.: +32 2 774 16 69
Internet:lco@eortc.be

Data Manager:
Marianne Pierart
Tel.: +32 2 774 16 03
Internet:mpi@eortc.be

All questions concerning membership in the cooperative groups should be addressed to the chairman and/or the secretary of the group.

18 Trial sponsorship/ financing

The sponsor of the study is the EORTC.
Ipsen Biothech contributes an educational grant to the EORTC.
The Director General of the EORTC Central Office / Data Center is:
Prof.Dr. Françoise Meunier
EORTC Central Office / Data Center
Avenue Mounier 83, bte 11
1200 BRUSSELS
BELGIUM
Tel.: +32 2 774 16 11
Fax: +32 2 772 35 45

19 Trial insurance

The EORTC insurance programme covers all patient entered in EORTC studies except patients from the USA and Canada.

19.1 Insurance within the European Union

When specific requirements are stated in the national laws of the E.U. countries, the insurance programme will take these requirements into account.
For countries where there are no specific requirements, the EORTC provides an insurance coverage which is valid for two years after a patient has completed the treatment strategy being studied by the research protocol. This insurance programme covers the EORTC as the promotor, the investigators and all local hospital staff.
For Germany, the specific requirements of the law (AMG) oblige the EORTC and its insurer to look at each individual protocol on a case by case basis before an insurance coverage can be provided.

19.2 Insurance outside the European Union

The EORTC insurance programme only covers claims against the EORTC ast the promotor in its role of coordinator of the research and not the investigators and local hospital staff.

20 Publication policy

The authors of the publications will be the study coordinator, a member of each centre having included at least 10 % of the evaluable patients for the abstracts and at least 5 % for the complete written publications (in sequence according to the number of fully evaluable patients) and the EORTC data managers and statisticians working on the study. No information can be used for the publication or oral presentation without the written agreement of the trial coordinator and the chairman of the group.

21 Administrative signature

Michel BOLLA , MD

22 LHRH analogue, drug delivery

Ipsen Biotech company will provide us with the drug Decapeptyl® (Triporéline) free of charge. The person who is in charge of the drug delivery is :

Pierrette CHEVREAU

BEAUFOUR-IPSEN-INDUSTRIE

(Service lots cliniques)

17-20, rue Ethe Virton

F - 28100 DREUX

Tel : + 33 (0)2 37 65 46 00 , Fax : + 33 (0)2 37 65 46 33

23 List of participants

Country	Institution/City
Belgium	AZVUB/Brussels.<br>Hôpital Saint Luc/Brussels
France	CHRU Hopital Michallon/Grenoble<br>CHRU Hopital J. Minjoz/Besançon<br>Centre G.F. Leclerc/Dijon<br>Fondation Bergonié/Bordeaux
Israel	Rambam Medical Center/Haifa,<br>Ichilov Hospital /Tel Aviv<br>Hadassah University Hospital/Jerusalem
Italy	Ospedale civile de Pordenone<br>San Raffaele Scientific Institute/Rome
The Netherlands	Academisch Medisch Centrum/Amsterdam<br>R.T.I.L /Heerlen,<br>Academisch Ziekenhuis/Maastricht
Malta	St Luke's Hospital/Malta
Norway	Haukeland Hospital/Bergen
Poland	University Hospital/Gdansk
Romania	Institute of Oncology/Cluj
Spain	Instituto Valenciano de Oncologia/Valencia
Switzerland	Ospedale san Giovanni/Bellinzona,<br>CHUV/ Lausanne.<br>Inselspital/ Bern
Turkey	Dokuz Eylul University of Medecine/Izmir.

24 References

1 Hanks GE, Krall JM, Hanlon AL, Asbell SO, Pilepich MV, and Owen JB. Patterns of care and RTOG studies in prostate cancer : long-term survival, hazard rate observations, and possibilities of cure. Int. J. Radiation Oncology Biol. Phys. 1993, 28, 39-45.

2 Leibel SA, Zelefsky MJ, Kutcher GJ, Burman CM, Mohan R, Mageras GS, Ling CC, and Fuks Z. The biological basis and clinical application of three-dimensional conformal therapy in carcinoma of the prostate. Sem. Oncol. 1994, 580-597.

3 Pilepich MV, Krall J, Al-Sarraf M, Roach M, Dogett RLS, Sause W, Lawton CA, Abrams RA, Rotman M, Rubin P, Shipley WU, Cox JD. A phase III trial of androgen suppression before and during radiation therapy for locally advanced prostatic carcinoma : preliminary report of RTOG protocol 8610. Proceedings of ASCO, 1993, 703, 229.

4 Pilepich MV, Caplan R, Byhardt RW, Lawton CA, Gallagher MJ, Mesic JB, Hanks GE, Coughlin CT, Porter A. Phase III trial of androgen suppression using goserelin in unfavorable prognosis carcinoma of the prostate treated with definitive radiotherapy. Proceedings of ASCO, 1995, 631, 239.

5 Bolla M. Controlled clinical trial in high metastatic risk carcinoma of the prostate comparing pelvic radiotherapy alone to pelvic radiotherapy plus LHRH analogue. Protocol number 22863. European Organization for Research and Treatment of Cancer.

6 Bolla M, Gonzalez D, Warde P, Dubois JB et al. Immediate hormonal therapy improves locoregional control and survival in patients with locally advanced prostate cancer. Results of a randomized phase III clinical trial of the EORTC radiotherapy and genito-urinary tract cancer cooperative groups. Proceedings of ASCO 1996;15:238 (abstract 591).

7 Labrie F, Belanger A, Simard J, Labrie C, and Dupont A. Combination therapy for prostate cancer. Cancer, 1993, 71, 3, 1059-1067.

8 Prostate Cancer Trialists Collaborative Group. Maximum androgen blockade in advanced prostate cancer : an overview of 22 randomized trials with 3283 deaths in 5720 patients. Lancet 1995, 346, 265-269.

9 Akakura K, Bruchovsky N, Goldenberg SL, Rennie PS, Buckley AR, and Sullivan LD. Effects of intermittent androgen suppression on androgen-dependent tumors. Apotosis and serum prostate-specific antigen. Cancer, 1993, 71, 2782-2790.

10 Schellhammer P.F., Vogelzang N.J., Roohollah Shafari, Norman L; Block, Mark S. Soloway et al. for the Casodex Study Group. Updated results of a randomized, dounle-blind trial in 813 previously untreated metastatic prostate cancer patients comparing the antiandrogens Casodex (Bicalutamide) and Eulexin (Flutamide) in combination with Luteinieing Hormone Releasing Hormone Analogue (LHRH-A). Proceedings of ASCO 1996;15:245 (abstract 619).

11 Kaplan E. L., Meier P. Non parametric estimation from incomplete observations. J. Am. Statit. Ass. 1958, 53, 457-481.

12 Mantel N. Evaluation of survival data and two new rank order statistics arising in its consideration. Cancer Chemother Rep, 1966, 50, 163-170.

13 Stiggelbout AM, Eijkemans MJC, Kiebert GM, Kievit J, Leer JWH, De Haes JCJM. The "utility" of the visual analog scale inmedical decision making and technology assessment. Int. J. Technol. Assessment in Health Care. 1996; 12 : 291-298.

Appendix 1:TNM classification of prostate cancer (UICC, 1992)

T - Primary tumor

Tx	Primary tumor cannot be assessed
T0	No evidence of primary tumor
**T1**	**Clinically inapparent tumor, not palpable or visible by imaging**
T1a	Tumor incidental finding in 5% or less of tissue resected
T1b	Tumor incidental finding in more than 5% of tissue resected
T1c	Tumor identified by needle biopsy (e.g., because of elevated PSA)
**T2**	**Tumor confined within prostate**
T2a	Tumor involves half of a lobe or less
T2b	Tumor involves more than half a lobe but not both lobes
T2c	Tumor involves both lobes
**T3**	**Tumor extends through the prostate capsule**
T3a	Unilateral extracapsular extension
T3b	Bilateral extracapsular extension
T3c	Tumor invades seminal vesicle(s)
**T4**	**Tumor is fixed or invades adjacent structures other than seminal vesicles**
T4a	Tumor invades any of the following : bladder neck, external sphincter, rectum
T4b	Tumor invades levator muscles and/or is fixed to pelvic wall

N- Regional lymph nodes

Nx	Regional lymph nodes cannot be assessed
N0	No regional lymph node metastasis
N1	Metastasis in a single lymph node, 2 cm or less in greatest dimension
N2	Metastasis in a single lymph node, more than 2 cm but not more 5 cm in greatest dimension, or multiple lymph nodes, none more than 5 cm in greatest dimension
N3	Metastasis in a lymph node, more than 5 cm in greatest dimension

M - Distant metastases

Mx	Presence of distant metastasis cannot be assessed
M0	No distant metastasis
M1	Distant metastasis
M1a	Nonregional lymph node(s)
M1b	Bone(s)
M1c	Other site(s)

Appendix 2: WHO performance status scale

GRADE 0 : Able to carry out all normal activity without restriction

GRADE 1 : Restricted in Physically strenuous activity but ambulatory and able to carry out light work.

GRADE 2 : Ambulatory and capable of all self-care but unable to carry out any work. Up and about more than 50% of waking hours.

GRADE 3 : Capable of only limited self-care, confined to bed or chair for more than 50% of waking hours.

GRADE 4 : Completely disabled; cannot carry on any self-care. Totally confined to bed or chair.

Appendix 3: Trial design

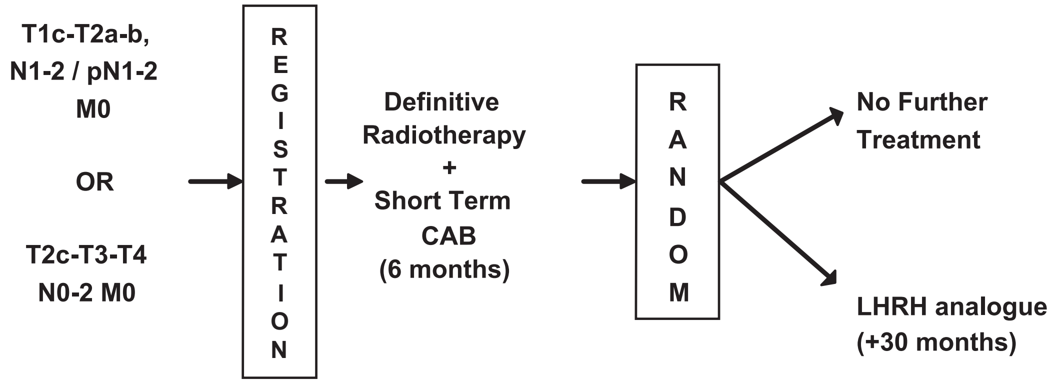

Appendix 4: Registration check list

Please connect to EuroCode direct line:+32-2-7720426/ PTT network:(206) 2215124
or through INTERNET:telnet "ecvax.eortc.be"
or phone the EORTC Data Center : +32-2-774 16 00

- WHO Performance status (0-4)
- Histologically proven diagnosis of adenocarcinoma of the prostate: 0= no, 1= yes
- Clinical T category : 1=T1c , 2= T2a, 3= T2b, 4= T2c, 5 = T3, 6 = T4
- Gleason (2-10)
- Clinical N category : 0= N0, 1= N1, 2= N2, 3= N3
- Pathological N category (after lymphadenectomy): 0= pN0, 1= pN1, 2= pN2, 8= not done

For boxes 7-10, please use code: 0= no, 1= yes

- Lymph node involvement to common iliac and/or periaortic lymph nodes
- Presence of distant metastases
- Other previous or concurrent malignancies, except adequately treated basal cell carc. of the skin
- Any type of treatment for prostate cancer except hormonotherapy given within 3 weeks prior to registration
- WBC **($x10^9$/l)**
- Platelets **($x10^9$/l)**
- Haemoglobin **(mmol/l)**
  or **(g/l)**
- PSA level (ng/ml)
- Upper Normal Laboratory Limit for PSA (ng/ml)
- Has informed consent according to local regulations been obtained ?: 0= no, 1= yes
- Affiliation of the member registering the patient: 1=RTX, 2=GU

INFORMATION ATTRIBUTED AT REGISTRATION

Patient sequence number

Date of registration *(dd/mm/yy)*

Appendix 4 (Continued): Randomization check list

Please connect to EuroCode direct line:+32-2-7720426/ PTT network:(206) 2215124
or through INTERNET:telnet "ecvax.eortc.be"
or phone the EORTC Data Center : +32-2-774 16 00

- Current WHO Performance status (0-4)

For boxes 2-6, please use code: 0= no, 1= yes

- Has the patient received 6 months of CAB?
- Is the patient in local progression after 6 months of CAB?
- Is the patient in regional progression after 6 months of CAB?
- Lymph node involvement to common iliac and/or periaortic lymph nodes?
- Presence of distant metastases?
- Current PSA level **(ng/ml)**

- Is patient able to understand and to fill in the quality of life questionnaire ? (0= no, 1= yes)
- ***!!!<u>Reminder</u>!!!*** *H*as baseline quality of life questionnaire been completed ? (0= no, 1= yes)

<u>INFORMATION ATTRIBUTED AT RANDOMIZATION</u> :

Treatment allocated at randomization

1 = No further treatment
2 = LHRH analogue (30 months)

Date of randomization *(dd/mm/yy)*

Appendix 5: Informed consent statement

Undersigned, Mr.

Name ....

Address ....

Zipcode/town..

Tel. nr. ....

Date of birth..

declares having been informed verbally and in writing on the trial.

The aims of the trial has been explained to me and I hereby declare to be willing to participate voluntarily in the trial.

Town:.. Date: :...

Signature patient : ....

Name physician in charge :..

Signature physician : ....

Appendix 6: Patient information sheet

The translation is under the responsability of each investigator and must be adapted to national regulation and be approved by local ethical committees.

Dear Sir,

Your physician told us that a prostate cancer has been diagnosed. This tumor appears to be amenable to curative therapy. The combination of an external irradiation of the pelvis and an adjuvant hormonal treatment of three years initiated at the onset of irradiation is the standard treatment for such a disease. However it remains under investigation to try to select the best treatment that provides the maximum of chances both for cure and for your quality of life.

Therefore the European Organization for Reseach and Treatment of Cancer (EORTC) has decided to conduct an international study comparing two treatment options. The aim is to know whether a short term hormonotherapy lasting 6 months, initiated at the onset of irradiation and followed for a short period after completion of radiotherapy, could provide the same results as those obtained by the same treatment which would be followed for 2. 5 years more by LHRH analogue. Such a short term adjuvant hormonotherapy, would be better tolerated and would provide the patient a better quality of life. LHRH analogues are potent analogues of naturally occuring LHRH which may offer a reversible medical castration; the side effects of medical castration are mainly symptoms of androgen withdrawal, such as impotence and hot flushes.

For your safety, you will be carefully checked during the study, accordingly to the study protocol previously approved by the Protocol Review Committee of the EORTC and subsequently by local Ethical Committee.

If you agree to participate in this study, you will be allocated after a pelvic external irradiation combined with a short term complete androgen blockade to one of the two treatments :
- no further treatment
- adjuvant hormonal treatment with LHRH analogues lasting 30 months. (LHRH analogues will be used as a subcutaneous monthly depot, or a 3 monthly depot preparation according to the opportunity of each institution).

Allocation to one of the two options will be done by a centralized computer system in Brussels. Your physician does not have any influence on allocation.

Participation to this study is completely voluntarily. The information obtained by your participation will be treated confidentially. As a participant to this trial, this information might be checked by the official regulatory authorities. In this situation, the anonymity will be guarenteed. You always have the right to withdraw from further participation if you cannot tolerate the treatment, for whatever reason. This will not have any consequence on your further treatment and you will continue the therapy with your physician.

You are kindly requested to give your written consent for this investigation. This insurance agreement of the study is applicable for all participants to this investigation.

Please do not hesitate to contact your attending physician.

Appendix 7: Flow chart of timing of controls and modality of follow-up (Months)*

	Clinical examination	Serum PSA	Quality of Life Question.	Late complications
**First Year**				
(6)	+	+	+	+
(12)	+	+	+	+
**Second Year**				
(18)	+	+		+
(24)	+	+	+	+
**Third Year**				
(30)	+	+		+
(36)	+	+	+	+
**Fourth Year**				
(42)	+	+		+
(48)	+	+	Health Thermometer	+
**Fifth Year**				
(54)	+	+		+
(60)	+	+	Health Thermometer	+
**Annually Thereafter**	+	+	Health Thermometer	+

* Chest X-ray, Technetium bone scan, CT scan of pelvis and abdomen are not mandatory each year ; they are required in presence of clinical and or biochimical (PSA) suspicion of progression.

Appendix 8: Toxicity

- Acute toxicity according to the CTC-NCIC toxicity scale

- Late toxicity modified EORTC/RTOG scale

0 = No symptoms

1 = Minor transient symptoms responding to single out-patient management

2 = Distressing, persistent or recurring symptoms requiring prolonged medical treatment occasionally necessitating brief hospitalization for diagnosis and/or minor surgical interventions (such as urethral dilatation)

3 = Complications requiring surgical procedure (laparotomy, colostomy, cystectomy) or continued hospitalization (over one month)

4 = Fatal complication

9 = Unknown

Appendix 9: Quality of life questionnaire

Appendix 10: Hospital visit form

Key information on hospitalization and outpatient visits should be filled in for the 30 MONTHS after randomization.

PLEASE GO TO A NEW LINE :

- **to report separately each kind of visit (even if the date is the same)**
- **each time a patient is hospitalized**
- **each time a patient is transferred to another department during hospitalization**
- **if the major reason for hospitalization changes**

|__|__| 01	Sequence of this form

Date of start of admission or visit (day/month/year)	Date of discharge (in case of hospitalization) (day/month/year)	Type of admission or visit (*)	Department (**)	Major reason for admission or visit (***)
__|__|____ 02	__|__|____ 03	__ 04	__ 05	__ 06
__|__|____ 07	__|__|____ 08	__ 09	__ 10	__ 11
__|__|____ 12	__|__|____ 13	__ 14	__ 15	__ 16
__|__|____ 17	__|__|____ 18	__ 19	__ 20	__ 21
__|__|____ 22	__|__|____ 23	__ 24	__ 25	__ 26
__|__|____ 27	__|__|____ 28	__ 29	__ 30	__ 31
__|__|____ 32	__|__|____ 33	__ 34	__ 35	__ 36
__|__|____ 37	__|__|____ 38	__ 39	__ 40	__ 41
__|__|____ 42	__|__|____ 43	__ 44	__ 45	__ 46
__|__|____ 47	__|__|____ 48	__ 49	__ 50	__ 51

Type of admission/visit (*)	Department (**)	Major reason for admission/visit (***)
1 = full hospitalization (night spent in hospital)	1 = Urology ward	1 = administration of hormonotherapy
2 = “one day” hospitalization (day care; no night spent in hospital)	2 = oncology ward	2 = radiotherapy
3 = outpatient visit	3 = radiotherapy ward	3 = surgery
8 = other :.........................	4 = intensive care unit	4 = observation / routine follow-up
9 = unknown	5 = isolation room	5 = progression of disease
	6 = general internal medicine	6 = side effects
	7 = outpatient clinic	7 = supportive therapy (other than 1-6)
	8 = other :.........................	8 = any other reason : ....
	9 = unknown	9 = unknown

Appendix 11: World medical association declaration of Helsinki

Recommendations guiding physicians in biomedical research involving human subjects

Adopted by the 18th World Medical Assembly
Helsinki, Finland, June 1964
and amended by the
29th World Medical Assembly
Tokyo, Japan, October 1975
35th World Medical Assembly
Venice, Italy, October 1983
and the
41st World Medical Assembly
Hong Kong, September 1989

Introduction

It is the mission of the physician to safeguard the health of the people. His or her knowledge and conscience are dedicated to the fulfilment of this mission.

The Declaration of Geneva of the World Medical Association binds the physician with the words, "The health of my patient will be my first consideration," and the International Code of Medical Ethics declares that, "A physician shall act only in the patient's interest when providing medical care which might have the effect of weakening the physical and mental condition of the patient."

The purpose of biomedical research involving human subjects must be to improve diagnostic, therapeutic and prophylactic procedures and the understanding of the etiology and pathogenesis of disease.

In current medical practice most diagnostic, therapeutic or prophylactic procedures involve hazards. This applies especially to biomedical research.

Medical progress is based on research which ultimately must rest in part on experimentation involving human subjects.

In the field of biomedical research a fundamental distinction must be recognised between medical research in which the aim is essentially diagnostic or therapeutic for a patient, and medical research, the essential object of which is purely scientific and without the implication of direct diagnostic or therapeutic value to the person subjected to the research.

Special caution must be exercised in the conduct of research which may effect the environment, and the welfare of animals used for research must be respected.

Because it is essential that the results of laboratory experiments be applied to human beings to further scientific knowledge and to help suffering humanity, the World

Medical Association has prepared the following recommendations as a guide to every physician in biomedical research involving human subjects. They should be kept under review in the future. It must be stressed that the standards as drafted are only a guide to physicians all over the world. Physicians are not relieved from criminal, civil and ethical responsibilities under the laws of their own countries.

I. Basic principles

1. *Biomedical research involving human subjects must conform to generally accepted scientific principles and should be based on adequately performed laboratory and animal experimentation and on a thorough knowledge of the scientific literature.*

2. *The design and performance of each experimental procedure involving human subjects should be clearly formulated in an experimental protocol which should be transmitted for consideration, comment and guidance to a specially appointed committee independent of the investigator and the sponsor provided that this independent committee is in conformity with the laws and regulations of the country in which the research experiment is performed.*

3. *Biomedical research involving human subjects should be conducted only by scientifically qualified persons and under the supervision of a clinically competent medical person. The responsibility for the human subject must always rest with a medically qualified person and never rest on the subject of the research, even though the subject has given his or her consent.*

4. *Biomedical research involving human subjects cannot legitimately be carried out unless the importance of the objective is in proportion to the inherent risk to the subject.*

5. *Every biomedical research project involving human subjects should be preceded by careful assessment of predictable risks in comparison with foreseeable benefits to the subject or to others. Concern for the interests of the subject must always prevail over the interests of science and society.*

6. *The right of the research subject to safeguard his or her integrity must always be respected. Every precaution should be taken to respect the privacy of the subject and to minimize the impact of the study on the subject's physical and mental integrity and on the personality of the subject.*

7. *Physicians should abstain from engaging in research projects involving human subjects unless they are satisfied that the hazards involved are believed to be predictable. Physicians should cease any investigation if the hazards are found to outweigh the potential benefits.*

8. *In publication of the results of his or her research, the physician is obliged to preserve the accuracy of the results. Reports of experimentation not in accordance with the principles laid down in this Declaration should not be accepted for publication.*

9. *In any research on human beings, each potential subject must be adequately informed of the aims, methods, anticipated benefits and potential hazards of the study and the discomfort it may entail. He or she should be informed that he or she is at liberty to abstain from participation in the study and that he or she is free to withdraw his or her consent to participation at any time. The physician should then obtain the subject's freely-given informed consent, preferably in writing.*

10. *When obtaining informed consent for the research project the physician should be particularly cautious if the subject is in a dependent relationship to him or her or may consent under duress. In that case the informed consent should be obtained by a physician who is not engaged in the investigation and who is completely independent of this official relationship.*

11. *In case of legal incompetence, informed consent should be obtained from the legal guardian in accordance with national legislation. Where physical or mental incapacity makes it impossible to obtain informed consent, or when the subject is a minor, permission from the responsible relative replaces that of the subject in accordance with national legislation. Whenever the minor child is in fact able to give consent, the minor's consent must be obtained in addition to the consent of the minor's legal guardian.*

12. *The research protocol should always contain a statement of the ethical considerations involved and should indicate that the principles enunciated in the present Declaration are complied with.*

II. Medical research combined with professional care(Clinical research)

1. *In the treatment of the sick person, the physician must be free to use a new diagnostic and therapeutic measure, if in his or her judgement it offers hope of saving life, re-establishing health or alleviating suffering.*

2. *The potential benefits, hazards and discomfort of a new method should be weighed against the advantages of the best current diagnostic and therapeutic methods.*

3. *In any medical study, every patient - including those of a control group, if any - should be assured of the best proven diagnostic and therapeutic method.*

4. *The refusal of the patient to participate in a study must never interfere with the physician-patient relationship.*

5. *If the physician considers it essential not to obtain informed consent, the specific reasons for this proposal should be stated in the experimental protocol for transmission to the independent committee (I, 2)*

6. *The physician can combine medical research with professional care, the objective being the acquisition of new medical knowledge, only to the extent that medical research is justified by its potential diagnostic or therapeutic value for the patient.*

III. Non-therapeutic biomedical research involving human subjects(Non-clinical biomedical research)

1. *In the purely scientific application of medical research carried out on a human being, it is the duty of the physician to remain the protector of the life and health of that person on whom biomedical research is being carried out.*

2. *The subjects should be volunteers - either healthy persons or patients for whom the experimental design is not related to the patient's illness.*

3. *The investigator or the investigating team should discontinue the research if in his/her or their judgement it may, if continued, be harmful to the individual.*

4. *In research on man, the interest of science and society should never take precedence over considerations related to the wellbeing of the subject.*
